# Cyclic increase in the histamine receptor H1-ADAM9-Snail/Slug axis as a potential therapeutic target for EMT-mediated progression of oral squamous cell carcinoma

**DOI:** 10.1038/s41419-025-07507-1

**Published:** 2025-03-20

**Authors:** Yi-Fang Ding, Kuo-Hao Ho, Wei-Jiunn Lee, Li-Hsin Chen, Feng-Koo Hsieh, Min-Che Tung, Shu-Hui Lin, Michael Hsiao, Shun-Fa Yang, Yi-Chieh Yang, Ming-Hsien Chien

**Affiliations:** 1 Department of Otolaryngology, School of Medicine, College of Medicine, Taipei Medical University, Taipei, Taiwan, ROC; 2 Department of Otolaryngology, Wan Fang Hospital, Taipei Medical University, Taipei, Taiwan, ROC; 3 Graduate Institute of Clinical Medicine, College of Medicine, Taipei Medical University, Taipei, Taiwan, ROC; 4 Department of Biochemistry and Molecular Cell Biology, School of Medicine, College of Medicine, Taipei Medical University, Taipei, Taiwan, ROC; 5 Department of Urology, School of Medicine, College of Medicine, Taipei Medical University, Taipei, Taiwan, ROC; 6 Department of Medical Education and Research, Wan Fang Hospital, Taipei Medical University, Taipei, Taiwan, ROC; 7 The Genome Engineering & Stem Cell Center, School of Medicine, Washington University, St. Louis, MO USA; 8 Department of Surgery, Tungs’ Taichung Metro Harbor Hospital, Taichung, Taiwan, ROC; 9 Department of Surgical Pathology, Changhua Christian Hospital, Changhua, Taiwan, ROC; 10 Department of Post-Baccalaureate Medicine, College of Medicine, National Chung Hsing University, Taichung, Taiwan, ROC; 11 Genomics Research Center, Academia Sinica, Taipei, Taiwan, ROC; 12 Institute of Medicine, Chung Shan Medical University, Taichung, Taiwan, ROC; 13 Department of Medical Research, Chung Shan Medical University Hospital, Taichung, Taiwan, ROC; 14 School of Oral Hygiene, College of Oral Medicine, Taipei Medical University, Taipei, Taiwan, ROC; 15 Department of Medical Research, Tungs’ Taichung MetroHarbor Hospital, Taichung, Taiwan, ROC; 16 TMU Research Center for Cancer Translational Medicine, Taipei Medical University, Taipei, Taiwan, ROC; 17 Pulmonary Research Center, Wan Fang Hospital, Taipei Medical University, Taipei, Taiwan, ROC; 18 Traditional Herbal Medicine Research Center, Taipei Medical University Hospital Taipei, Taipei, Taiwan, ROC

**Keywords:** Oral cancer, Metastasis

## Abstract

The intricate involvement of the histaminergic system, encompassing histamine and histamine receptors, in the progression of diverse neoplasias has attracted considerable scrutiny. Histamine receptor H1 (HRH1) was reported to be overexpressed in several cancer types, but its specific functional implications in oral squamous cell carcinoma (OSCC) predominantly remain unexplored. Our findings indicate that dysregulated high levels of HRH1 were correlated with lymph node (LN) metastasis and poor prognoses in OSCC patients. We identified a disintegrin and metalloprotease 9 (ADAM9) as a critical downstream target of HRH1, promoting protumorigenic and prometastatic characteristics both in vitro and in vivo. Molecular investigations revealed that the cyclic increase in the HRH1-ADAM9-Snail/Slug axis promoted progression of the epithelial-to-mesenchymal transition (EMT). Clinical analyses demonstrated significant correlations of HRH1 expression with ADAM9 and with EMT-related markers, with elevated ADAM9 also associated with LN metastasis in OSCC patients. Regarding therapeutic aspects, we discovered that activated STAT3 acts as a compensatory pathway for the long-term HRH1 signaling blockade in OSCC cells. Combining inhibition of HRH1 and STAT3 using their respective inhibitors or short hairpin (sh)RNAs enhanced the tumor-suppressive effects compared to HRH1 inhibition/depletion alone in OSCC cells and a xenograft model. In summary, HRH1 has emerged as a valuable biomarker for predicting OSCC progression, and combined targeting of HRH1 and STAT3 may represent a promising strategy for preventing OSCC progression.

## Introduction

The majority of head and neck cancers originate from squamous cells located on the mucosal epithelium of the head and neck. These cancers are referred to as head and neck squamous cell carcinoma (HNSCC), ranking as the sixth most frequently diagnosed cancer globally [1]. Oral squamous cell carcinoma (OSCC) represents a significant subset of HNSCC cases [2]. Most types of HNSCC, especially OSCC, are easily noticeable due to their localized presence. Although an OSCC diagnosis typically relies on histological evaluation of tissue biopsies, this approach often results in delayed diagnoses. Therefore, certain noninvasive diagnostic techniques, such as those targeting genetic alterations and aberrant protein activation or expression, were reported to be useful in predicting disease progression. For example, elevated hypermethylation levels of *NID2* and *HOXA9* in salivary cells have shown potential for early oral cancer detection [3]. Roi and colleagues identified over 100 molecules in saliva, including several promising cytokines, such as IL6 and IL8, that could serve as biomarkers for oral cancer [4]. In addition, the loss of several tumor-suppressor genes, including *CDKN2A*, *ARF*, and *TP53*, was reported to occur during the progression from normal mucosa to dysplasia [5, 6]. Additionally, ~80–90% of patients with HNSCC exhibit overexpression of the epidermal growth factor receptor (EGFR), with higher EGFR expression often associated with poorer prognostic outcomes [7]. Although some molecular targets may be implicated in HNSCC diagnosis or treatment, effective therapeutic approaches in clinical practice remain limited. For instance, cetuximab, a monoclonal antibody targeting the EGFR, and immune checkpoint inhibitors such as nivolumab and pembrolizumab have been approved for HNSCC treatment. However, clinical benefits are observed in only ~15–20% of HNSCC patients treated with these agents, and response rates to these treatments remain low in patients with advanced (metastatic) HNSCC [8, 9]. Hence, it is imperative to identify new druggable targets that can be utilized for early detection or optimal treatment of metastasis.

Histamine, a bioactive amine (2-[4-imidazole]-ethylamine), mediates diverse immune responses via four G-protein-coupled receptor (GPCR) subtypes: histamine receptor (HR) H1 (HRH1), HRH2, HRH3, and HRH4 [10]. HRH1 was the first receptor identified and targeted for treating allergic inflammation. Although the association between allergies and cancer risk remains contentious [11, 12], recent studies highlighted the significant involvement of histamine and specific HRs in various aspects of cancer development, including growth and metastasis [13]. For example, cancer cells often exhibit increased activity of a histamine-synthesizing enzyme called L-histidine decarboxylase (HDC), leading to elevated histamine levels in patients with breast cancer and melanoma [14–16]. HRH1 overexpression in hepatocellular carcinoma (HCC) was shown to promote tumor metastasis by inducing matrix metalloproteinase (MMP)-2 [17], and treatment with HRH1 antagonists was reported to decrease the HCC risk in patients with hepatitis B virus (HBV), HCV, or dual infection [18]. Inhibiting HRH1 in basal and human EGFR 2 (HER2)-targeted therapy-resistant breast cancer cells can inhibit proliferation, activate mitochondrial apoptotic pathways, and enhance the effectiveness of HER2-targeted therapy [19]. Higher HRH1 expression by tumor-associated macrophages (TAMs) was shown to accelerate tumor growth by suppressing the cluster of differentiation-positive (CD8+) T-cell activity, and antihistamine treatment was shown to augment the effectiveness of immune checkpoint blockade (ICB) therapies in lung cancer and melanoma [20]. Additionally, blocking HRH1 in colon cancer cells enhanced the therapeutic effects of radiation [21]. Although HRH1 expression was reported to correlate with poor prognoses in OSCC patients [22], our understanding of the mechanism by which HRH1 contributes to OSCC development remains limited.

In the present study, we explored the impact and mechanism of HRH1 in human OSCC. Our findings revealed a significant elevation of HRH1 levels in human HNSCC and OSCC tissues, with its upregulation correlating with disease progression and serving as an independent prognostic factor in OSCC patients. HRH1-knockdown (KD) suppressed OSCC cell proliferation, colony formation, and motility in vitro while inhibiting OSCC growth and cervical lymph node (LN) metastasis in mice. Mechanistically, we demonstrate that the cyclic upregulation of the HRH1-ADAM9-Snail/Slug axis promotes epithelial-to-mesenchymal transition (EMT)-mediated OSCC progression. Moreover, we uncovered that activated signal transducer and activator of transcription 3 (STAT3) may act as a compensatory pathway in response to HRH1 depletion in OSCC cells. Regarding therapeutic implications, the combined inhibition of HRH1 and STAT3 enhanced tumor-suppressive effects compared to HRH1 targeting alone in OSCC cells and a xenograft model. Thus, HRH1 has emerged as a valuable biomarker for predicting OSCC progression, and the combined targeting of HRH1 and STAT3 may offer a promising strategy for preventing OSCC progression.

## Results

### HRH1 is elevated in HNSCC specimens and is correlated with tumor progression and a poor prognosis

**Fig. 1:**
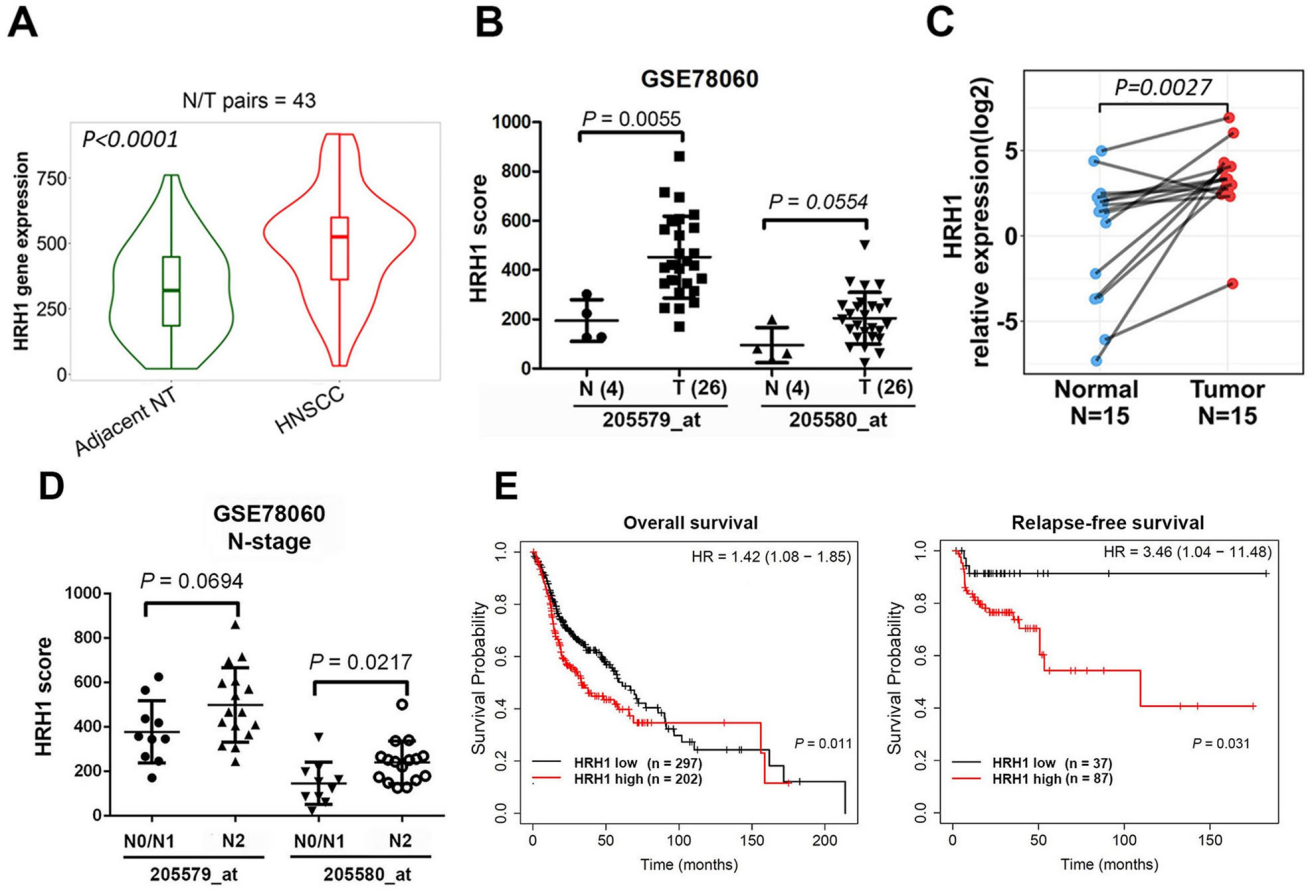
HRH1 is elevated in OSCC tissues and correlated with lymph node metastasis and poor prognosis. HRH1 mRNA expression levels in paired adjacent and unpaired normal and tumor tissues respectively derived from patients with head and neck squamous cell carcinoma (HNSCC) (**A**) and OSCC (**B**). The Mann–Whitney *U*-test was performed to evaluate the statistical differences. Data for HNSCC were retrieved from TNMplot, while data for OSCC were retrieved from GEO (GSE78060). **C** Related mRNA expression of *HRH1* in Taiwanese OSCC and adjacent normal tissues evaluated by an RT-qPCR. A paired *t-*test was performed to assess the statistical differences. **D** Expression levels of *HRH1* mRNA in OSCC with different N (node) stages were determined by analyzing the GSE78060 dataset. **E** Correlations of HRH1 expression with overall survival and relapse-free survival in patients with HNSCC as determined using a Kaplan–Meier plotter database. Gene expressions were dichotomized into high and low values using the best cutoff value. *p* < 0.05 was considered to indicate a statistically significant difference. HR hazard ratio.

To investigate the clinical relevance of HRH1 in HNSCC, we initially assessed *HRH1* gene expression levels between tumor and noncancerous tissues using three online databases: TIMER2.0 [23], TNMplot [24], and GEO [25]. As depicted in Fig. S1, analysis of different cancer cohorts from TCGA revealed elevated *HRH1* levels across various cancer types, with a particularly significant increase observed in HNSCC. Furthermore, we observed significantly higher *HRH1* levels in HNSCC tissues compared to adjacent normal tissues (Fig. 1A). Additionally, analysis of two HRH1 probes (205579_at and 205580_at) in an OSCC cohort from the GSE78060 dataset demonstrated higher *HRH1* levels in tumor tissues compared to normal tissues (Fig. 1B). Consistently, in the Taiwanese population, we observed significantly elevated *HRH1* messenger (m)RNA levels in OSCC patient samples compared to paired normal tissue samples (Fig. 1C).

We conducted further analysis on correlations of *HRH1* expression with patients’ clinicopathological characteristics and survival rates. A positive correlation of *HRH1* levels with LN metastasis was observed in OSCC patients retrieved from the GSE78060 dataset (Fig. 1D). Higher *HRH1* levels also showed a trend toward larger tumor sizes (Fig. S2A). Kaplan–Meier plots revealed that HNSCC and OSCC patients with high *HRH1* expression, respectively, retrieved from TCGA and GSE31056 had poorer OS or RFS rates (Figs. 1E and S2B). Additionally, a univariate Cox regression analysis was conducted to examine the prognostic significance of the clinicopathologic variables in the same HNSCC cohort. Results indicated that *HRH1* expression hazard ratio (HR), 1.369; *p* = 0.024), LN metastasis (HR, 1.861; *p* < 0.001), and tumor size (HR, 1.983; *p* < 0.001) were all associated with adverse impacts on OS. To assess the independent contribution of *HRH1* to survival, a multivariate analysis was performed. Results revealed that the *HRH1* expression level could serve as an independent prognostic factor for OS in HNSCC patients (Table 1). Taken together, these findings suggest that *HRH1* acts as an oncogene, promoting the malignancy of HNSCC.

**Table 1:** Cox univariate regression analysis of prognostic factors and HRH1 expression for overall survival in 374 HNSCC patients.

Variable	Comparison	HR (95% CI)	*p* value
Cox univariate analysis (OS)			
Age	≥60 vs. <60	1.254 (0.949–1.656)	0.111
Gender	Male vs Female	0.753 (0.563–1.007)	0.056
Stage	3, 4 vs. 1, 2	1.867 (1.259–2.768)	**0.002**
T status	3, 4 vs. 1, 2	1.983 (1.434–2.743)	**<0.001**
N status	1, 2, 3 vs. 0	1.861(1.335–2.594)	**<0.001**
HRH1	High vs. Low	1.369 (1.042–1.798)	**0.024**
Cox multivariate analysis (OS)			
Age	≥60 vs. <60	1.243 (0.896–1.724)	0.194
Gender	Male vs. Female	0.784 (0.553–1.111)	0.171
Stage	3, 4 vs. 1, 2	1.121 (0.518–2.427)	0.772
T status	3, 4 vs. 1, 2	1.978 (1.207–3.241)	**0.007**
N status	1, 2, 3 vs. 0	1.736 (1.175–2.564)	**0.006**
HRH1	High vs. Low	1.391 (1.014–1.909)	**0.041**

*HR* hazard ratio, *CI* confidence interval, *T* tumor, *N* node.

Bold values indicates statistically significant *p* values less than 0.05.

### HRH1 promotes in vitro tumorigenesis and an invasive phenotype of HNSCC cells through both histamine-dependent and -independent mechanisms

**Fig. 2:**
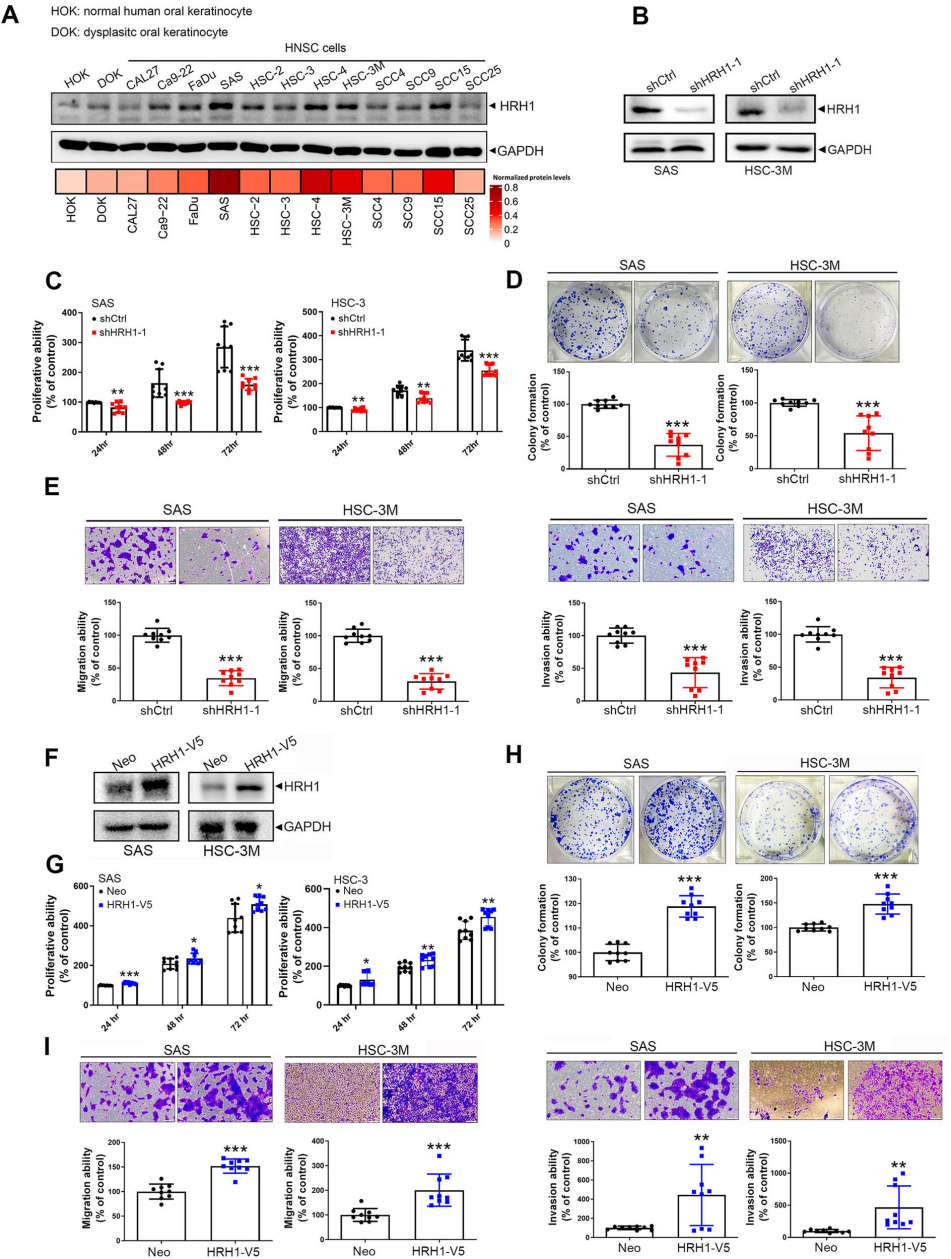
HRH1 promotes cell proliferative, colony-forming, migratory, and invasive abilities in OSCC cells. **A** Estimation of HRH1 protein levels in oral keratinocytes and head and neck squamous cell carcinoma (HNSCC) cell lines by Western blotting. GAPDH was used as an internal loading control (upper panel). Quantitative data were obtained by measuring the signal density using ImageJ software and were then normalized with GAPDH (lower panel). **B** The efficacy of HRH1-knockdown in SAS and HSC-3M cells was verified by Western blotting. The MTS (**C**) and colony-forming (**D**) assays of SAS and HSC-3M cells after knockdown of HRH1. **E** Results of migration (left panel) and Matrigel-invasion (right panel) assays in SAS and HSC-3M cells after knockdown of HRH1. **F** HRH1 was overexpressed in SAS and HSC-3M cells, as determined by Western blotting. Proliferation rates (**G**) and colony-formation capacities (**H**) of HRH1-overexpressing SAS and HSC-3M cells were respectively determined using MTS and colony-forming assays. **I** Migratory (left panel) and invasive (right panel) abilities of HRH1-overexpressing SAS and HSC-3M cells were respectively evaluated by migration and Matrigel-invasion assays. Data are shown as the mean ± standard deviation (SD), *n* = 3, two-tailed Student’s *t*-test. *p < 0.05, **p < 0.01, ***p < 0.001.

Similar to results observed in clinical samples, we observed significantly elevated protein levels of HRH1 in most of the HNSCC/OSCC cell lines included in our study (i.e., Ca9-22, FaDu, SAS, HSC-2, HSC-3, HSC-4, HSC-3M, SCC4, SCC9, and SCC15) compared to normal or dysplastic oral keratinocytes (HOK and DOK; Fig. 2A). To further determine the functional role of HRH1 in facilitating the progression of HNSCC, we evaluated the effect of HRH1 expression on cell behaviors, including cell growth and metastasis, two fundamental steps of tumor progression.

First, we utilized a lentiviral-based RNA-KD approach to establish stable KD of HRH1 in SAS and HSC-3M OSCC cells (Fig. 2B). Effects of HRH1 on short-term (24–72 h) and long-term (8 days) growth of both OSCC cells were respectively assessed by MTS and colony-formation assays. We observed that HRH1-KD significantly suppressed cell proliferation (Fig. 2C) and clonogenicity (Fig. 2D). Additionally, HRH1-KD also remarkably reduced migration and invasion, two key elements of cancer metastasis, in both OSCC cell lines (Fig. 2E). In contrast to the HRH1-KD in OSCC cells, we also established stable HRH1-overexpressing SAS and HSC-3M cells (Fig. 2F) and found that HRH1 overexpression caused opposite effects (Fig. 2G–I).

In addition to HRH1 overexpression, histamine, an HRH1 agonist, was reported to be elevated in various cancer types and to act as an autocrine growth factor in promoting cancer progression [17, 26]. To further explore the potential effect of histamine on HRH1-regulated OSCC progression, SAS and HSC-3M cells were treated with 1 μM histamine. As depicted in Fig. S3A and S3B, histamine treatment significantly increased the colony-forming, migratory, and invasive abilities of both OSCC cell lines. Moreover, histamine treatment-induced increases in migration and invasion were significantly reversed under HRH1-KD in SAS cells (Fig. S4). These findings collectively indicate that histamine promotes the growth and metastasis of OSCC cells by acting on HRH1, while HRH1 may also have a histamine-independent role in promoting OSCC progression.

### Analysis of potential molecular mechanisms regulated by HRH1 in HNSCC progression

**Fig. 3:**
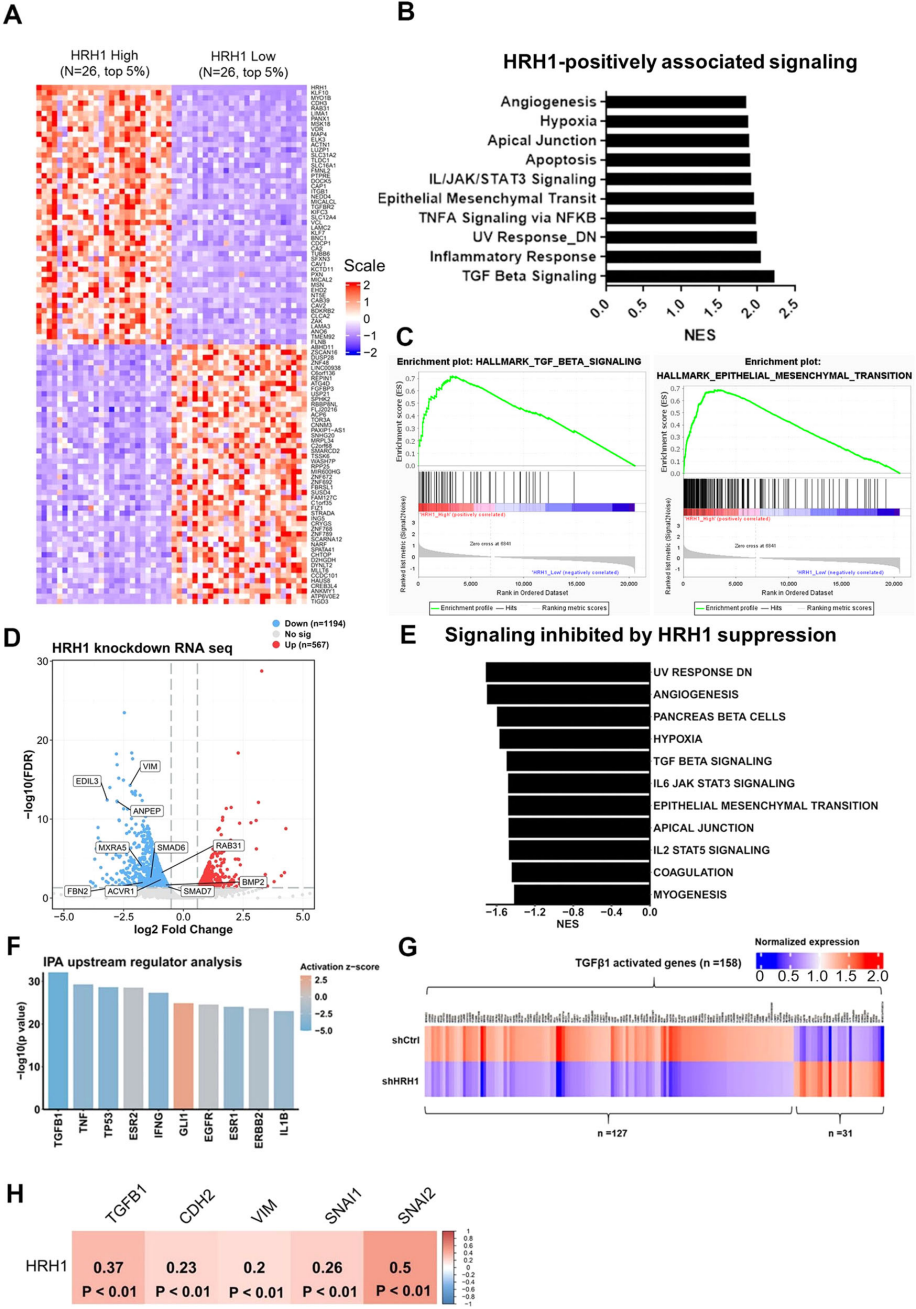
Analysis of potential HRH1-regulated pathways involved in head and neck squamous cell carcinoma (HNSCC) progression. **A** Heatmap displaying expressions of differentially expressed genes among the top 5% of TCGA-HNSCC patients with the highest or lowest HRH1 expression. **B** Horizontal bar plot illustrating the top 10 enriched gene sets in the HRH1-high group, utilizing the gene set database, h.all.v7.5.symbols.gmt [Hallmark]. **C** Representative enrichment plots for transforming growth factor (TGF)-β signaling and the EMT, which are significantly positively associated with high HRH1 expression. The EMT gene set was derived from h.all.v7.5.symbols.gmt [Hallmark]. **D** Volcano plot illustrates genes that are downregulated (FC of <0.7, FDR of <0.05; 1194 genes represented by blue dots) and upregulated (FC of >1.5, FDR of <0.05; 567 genes represented by red dots) in SAS/shHRH1 cells. **E** GSEA of altered genes in shHRH1 versus shCtrl groups. **F** Ingenuity pathway analysis (IPA) of shHRH1 versus shCtrl in SAS cells for the top 10 significant upstream regulators of SAS/shHRH1 cells. **G** Heatmap indicates highly positive associations between HRH1 expression and TGF-β1-regulated genes in SAS cells. **H** Correlation plot demonstrates correlations between *HRH1* gene expression and TGF-β as well as EMT markers. RNA sequencing data from TCGA-HNSCC patients were utilized for this analysis, with correlation coefficients and *p* values evaluated through a Pearson correlation analysis. TCGA the cancer genome atlas, EMT epithelial-to-mesenchymal transition, GSEA gene set enrichment analysis, FC fold change, FDR false discovery rate.

To explore underlying molecular mechanisms of HRH1 in promoting progression of HNSCC, we initially employed a GSEA to compare gene profiles between patients with the top 5% highest (*N* = 26) or lowest 5% (*N* = 26) HRH1 expression levels in TCGA HNSCC cohort. The heatmap in Fig. 3A visualizes the 50 most highly ranked genes in both the high- and low-HRH1 level groups (Table S1). Upon analyzing enriched signature gene sets in the high-HRH1 group, we identified significant associations between HRH1 and key signaling pathways (Fig. 3B), including EMT and TGF-β signaling (Fig. 3C), both known to play critical roles in HNSCC progression [27]. To corroborate our analysis of clinical data from TCGA cohort, we conducted RNA-Seq on SAS/shHRH1 and SAS/shCtrl cells. Results showed that 1194 genes were downregulated and 567 genes were upregulated in SAS/shHRH1 cells compared to control cells (Fig. 3D). Subsequently, the altered genes identified from RNA-Seq were subjected to a GSEA (Fig. 3E), revealing similar pathway alterations as observed in Fig. 3B. Additionally, an IPA of RNA-Seq data yielded consistent results, highlighting the significance of TGF-β1 signaling in cells with HRH1 manipulation (Fig. 3F and Table S2). Notably, a majority (>80%) of genes activated by TGF-β1 signaling were found to be suppressed in HRH1-KD cells (Fig. 3G). Furthermore, a positive correlation between HRH1 and TGF-β1, as well as mesenchymal markers including N-cadherin (CDH2), vimentin (VIM), Snail (SNAI1), and Slug (SNAI2), was observed in TCGA HNSCC cohort (Fig. 3H).

### HRH1 facilitates the progression of OSCC by promoting the TGF-β-mediated EMT process

**Fig. 4:**
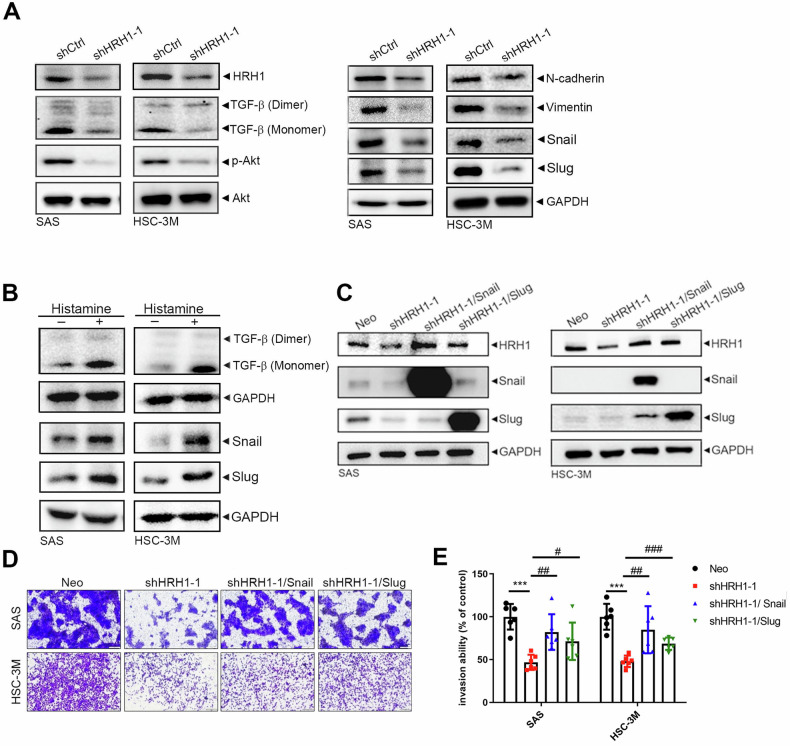
HRH1 facilitates OSCC progression via promoting the TGF-β-mediated EMT process. **A** Protein expression levels of activated TGF-β, Akt, and EMT-related markers such as N-cadherin, vimentin, Snail, and Slug in SAS (left panel) and HSC-3M (right panel) cells after knockdown of HRH1 as detected by Western blotting. **B** Treatment of SAS and HSC-3M cells with histamine (1 µM) for 24 h and the activated TGF-β and EMT markers were determined by Western blotting. **C**–**E** A plasmid expressing Slug or Snail was transfected into HRH1-knockdown SAS and HSC-3M cells as indicated, and a Western blot analysis was conducted to detect expression levels of HRH1, Snail, and Slug (**C**). Additionally, a Matrigel invasion assay was performed. Representative images (**D**) and quantification (**E**) of invading OSCC cells are shown. Differences are presented as the mean ± standard deviation (SD). *n* = 2; * *p* < 0.05, *** *p* < 0.001, com*p*ared to the control group; ^#^ *p* < 0.05, ^##^ *p* < 0.01, ^###^ *p* < 0.001, compared to the HRH1-knowdown only group.

In OSCC, TGF-β signaling was implicated in the EMT through Snail and Slug, leading to upregulation of matrix metalloprotease (MMP)-9 levels, thereby promoting cell invasion [28, 29]. Herein, we observed that activated TGF-β and its downstream signal, p-Akt, along with critical mesenchymal-related markers (N-cadherin, vimentin, Snail, and Slug), were all downregulated in HRH1-KD SAS and HSC-3M cells (Fig. 4A). Conversely, histamine treatment induced upregulation of activated TGF-β, Snail, and Slug (Fig. 4B). Next, to determine whether Snail family members play critical roles in HRH1-modulated cell motility, we overexpressed Snail or Slug in both OSCC cell lines to reverse HRH1-KD-mediated downregulation of the Snail family (Fig. 4C). Inhibition of cell invasion imposed by HRH1 depletion was also significantly reversed (Fig. 4D, E). Taken together, these data indicate that HRH1 modulates OSCC progression via inducing the TGF-β-Snail/Slug-mediated EMT. Additionally, we surprisingly found that downregulation of HRH1 protein and mRNA imposed by HRH1-KD was also reversed by overexpressing Snail or Slug in OSCC cells (Figs. 4C and S5), suggesting that TGF-β-Snail/Slug signaling induced by HRH1 may exhibit positive feedback regulation on HRH1 in OSCC cells.

### ADAM9 expression correlates with HRH1 in OSCC tissues and is essential for the HRH1-induced TGF-β-mediated EMT and cell motility in OSCC cells

**Fig. 5:**
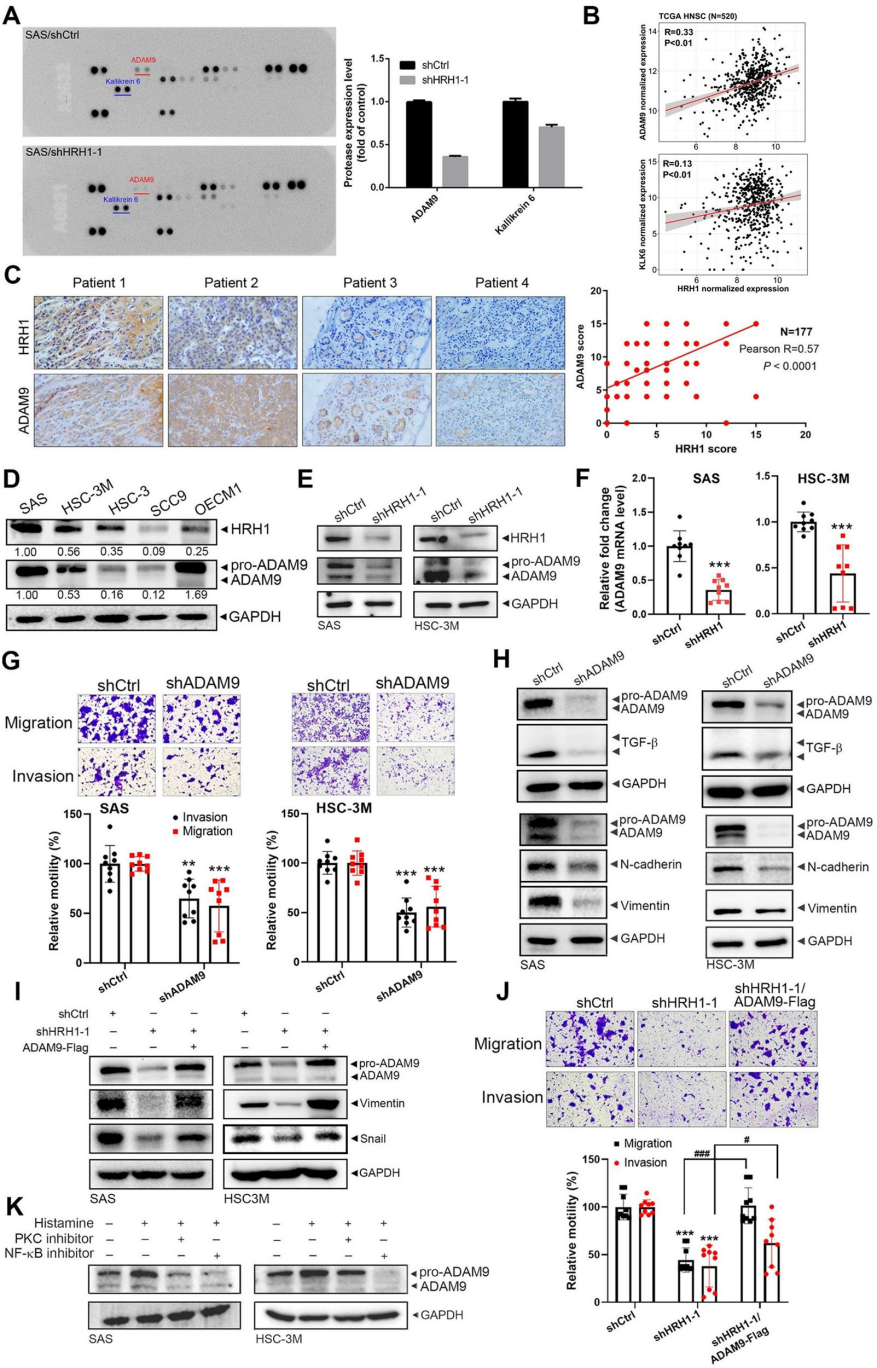
ADAM9 is crucial for HRH1-induced EMT-mediated progression of OSCC. **A** Protease expressions were compared in SAS/shCtrl and SAS/shHRH1 cell lysates using an antibody array (R&D Systems) that detected 34 proteases. Representative blots are shown on the left, and quantification was conducted using a densitometer on the right. Data are presented as the mean ± standard deviation (SD) (*n* = 2). **B** Correlations of *HRH1* gene expression with the *ADAM9* and kallifrein-6 (*KLK6*) genes were demonstrated in a correlation plot using RNA sequencing data of TCGA head and neck squamous cell carcinoma (HNSCC) patients. Correlation coefficients and *p* values were evaluated by a Pearson correlation analysis. **C** IHC staining was performed for HRH1 and ADAM9 on serial sections of OSCC specimens (*n* = 177) from Taiwanese patients. Representative images of IHC staining for HRH1 and ADAM9 in human OSCC tissues are shown on the left. Pearson correlation analysis indicated a positive correlation between HRH1 and ADAM9 expression, as depicted on the right. **D** Estimation of HRH1 and ADAM9 protein levels in a set of OSCC cell lines by Western blotting. Quantitative results of HRH1 and ADAM9 protein levels were normalized to GAPDH levels. ADAM9 protein and mRNA levels were respectively determined by Western blotting (**E**) and an RT-qPCR (**F**) in SAS and HSC-3M cells after knockdown of HRH1. **G** Migratory and invasive abilities of SAS and HSC-3M cells with ADAM9-knockdown were respectively determined by migration and Matrigel-invasion assays. **H** Protein expression levels of ADAM9, TGF-β, and EMT-related markers in SAS (left panel) and HSC-3M (right panel) cells after knockdown of ADAM9 were detected by Western blotting. An ADAM9-expressing plasmid was transfected into HRH1-knockdown SAS and HSC-3M cells as indicated and subjected to Western blotting (**I**) and migration and Matrigel-invasion assays (**J**). SAS and HSC-3M cells were pretreated with or without 5 μM chelerythrine or 100 nM EVP4593 for 1 h followed by 1 μM histamine treatment for an additional 24 h. Expression levels of cleaved ADAM9 were determined by Western blotting. **F**, **G**, **J** Data are shown as the mean ± standard deviation (SD), *n* = 3, two-tailed Student’s *t*-test. * *p* < 0.05, ** *p* < 0.01, *** *p* < 0.001, compared to the control group; ^#^ *p* < 0.05, ^##^ *p* < 0.01, compared to the HRH1-knowdown only group. IHC, immunohistochemistry.

MMPs were reported to play crucial roles in the TGF-β-induced EMT during tumor progression [30]. Next, we performed proteomic screening using a human protease array (Fig. 5A, left panel) to investigate the effects of HRH1 on protease expressions. Among 35 proteases, we found two proteases, ADAM9 and kallikrein 6 (KLK6), that were downregulated in HRH1-depleted SAS cells compared to control cells (Fig. 5A, right panel). Moreover, compared to KLK6, ADAM9 gene expression exhibited a higher correlation with HRH1 in HNSCC samples retrieved from TCGA (Fig. 5B). Similar to HRH1, elevated ADAM9 transcripts were also observed in HNSCC samples (Fig. S6A) and OSCC samples (GSE78060) (Fig. S6B) and were correlated with LN metastasis of OSCC (Fig. S6C). In addition to ADAM9 mRNA levels, we actually observed a significant correlation (correlation coefficient *r* = 0.57, *p* < 0.0001) between ADAM9 and HRH1 protein levels in OSCC samples retrieved from a Taiwanese population (Fig. 5C). Our previous study confirmed the expression levels of HRH1 in a set of OSCC cell lines (SAS, HSC-3M, HSC3, SCC9, and OECM1) [31]. Consistent with observations from clinical specimens, a correlation between ADAM9 (both in proform and active form) and HRH1 protein levels was also noted in these OSCC cell lines (Fig. 5D). We further validated the modulation of ADAM9 expression by HRH1 in OSCC cells, observing decreased levels of both ADAM9 protein and mRNA after HRH1-KD in SAS and HSC-3M cells (Fig. 5E, F). In contrast, the Western blot analysis using different ADAM9-specific antibodies all revealed upregulation of ADAM9 in HRH1-overexpressing HSC-3M cells (Fig. S7A). To assess the functional role of ADAM9 in OSCC, we knocked down ADAM9 in the SAS and HSC-3M cell lines, resulting in significantly reduced migratory and invasive abilities compared to control cells (Fig. 5G). Additionally, ADAM9-KD led to the downregulation of TGF-β and mesenchymal-related markers (N-cadherin and vimentin) (Fig. 5H). Further investigation into the role of ADAM9 expression in the HRH1-promoted EMT and cell mobility involved re-expressing ADAM9 in HRH1-KD SAS and HSC-3M cells through transfection with an ADAM9-expressing plasmid (Fig. 5I). This re-expression dramatically reversed the suppression of vimentin, Snail, and cell migratory/invasive abilities induced by HRH1-KD in both OSCC cell lines (Figs. 5I, J and S8). Taken together, these results suggest the dependence on ADAM9 of the HRH1-induced TGF-β-mediated EMT and cell motility in OSCC cells.

In addition to the histamine-independent role of HRH1 in inducing ADAM9 expression, treatment of SAS cells with histamine also resulted in concentration-dependent upregulation of ADAM9 (Fig. S7B). Co-IP experiments revealed no association between ADAM9 and HRH1 in SAS cells (Fig. S7C), suggesting that the histamine-HRH1 axis indirectly upregulates ADAM9 expression in OSCC cells. To further elucidate downstream signals involved in histamine-HRH1 axis-induced ADAM9 expression, we utilized inhibitors of protein kinase C (PKC) (chelerythrine) and nuclear factor (NF)-κB (EVP4593), two major signaling pathways activated by HRH1 [13, 32]. Our data revealed that inhibition of PKC and NF-κB reversed histamine-induced upregulation of ADAM9 in SAS and HSC-3M cells (Fig. 5K), suggesting coordinated regulation of ADAM9 expression under HRH1 activation by the PKC and NF-κB pathways.

### HRH1 depletion suppresses tumorigenicity and cervical LN metastasis of OSCC in an orthotopic mouse model

**Fig. 6:**
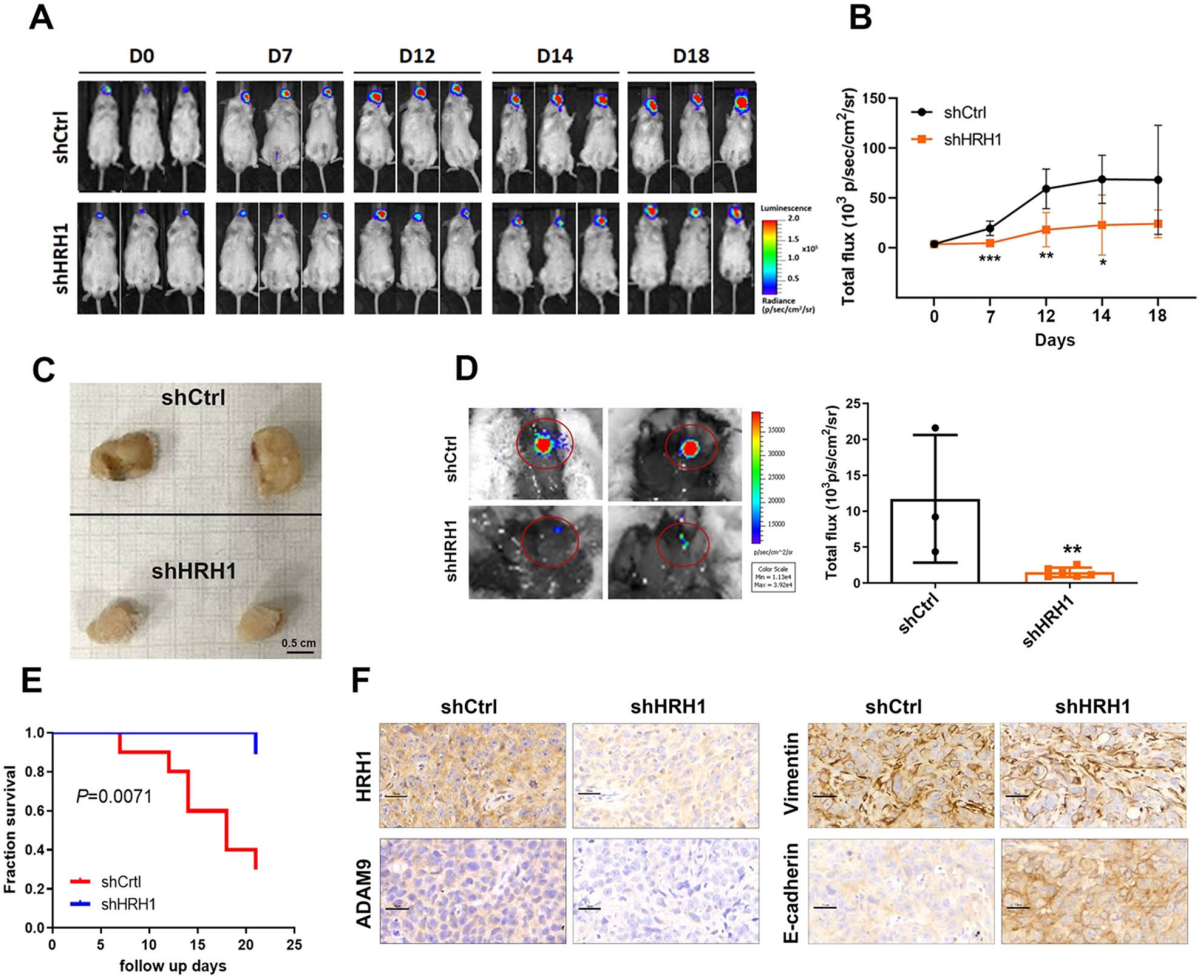
HRH1-knockdown suppresses tumor growth and cervical lymph node (LN) metastasis of OSCC in orthotopic mouse models. **A**, **B** Luciferase-tagged SAS/shCtrl or SAS/shHRH1 cells were orthotopically injected into NOD/SCID mice (*n* = 6). Luciferase activity images are shown in (**A**). The quantitative analysis of Xenogen imaging signal intensity (photons/s/cm^2^/sr) is shown in (**B**). *p < 0.05, **p < 0.01, ***p < 0.001, compared to the control group. **C** Gross appearance of orthotopic tumors 21 days after injecting SAS/shCtrl or SAS/shHRH1 cells. Scale bar = 0.5 cm. **D** Cervical LN metastasis (red circle indicated) was bioluminescent imaging at the end of the study (left panel) with the mean signal for each group indicated (right panel). **p < 0.01, compared to the control group. **E** Kaplan–Meier survival curves for mice injected with HRH1-depleted SAS cells or control cells (*n* = 10). *p* values were analyzed by a log-ra*n*k test. **F** SAS xenografts with or without HRH1-knockdown were isolated to detect expressions of HRH1, ADAM9, vimentin, and E-cadherin by IHC staining. Original magnification, 400 × Scale bar, 30 µm.

We further investigated the in vivo impact of HRH1 on OSCC progression by establishing an orthotopic OSCC-bearing animal model. This involved transplanting luciferase-tagged cells, either SAS-shCtrl-Luc or SAS-shHRH1-Luc, into NOD-SCID mice. Tumor progression was monitored twice weekly via bioluminescence imaging (Fig. 6A). In vivo photon emission detection revealed that HRH1-KD led to attenuated tumor growth compared to the control group (Fig. 6B). Upon sacrifice of the mice at the end of the experiment (21 days post-cell injection), visible tumor masses (Fig. 6C) were smaller in the HRH1-KD group than in the control group. Moreover, HRH1-KD significantly impeded the formation of spontaneous metastasis, as evidenced by ex vivo photon images showing lower photon intensity in cervical LN of SAS-shHRH1-Luc-injected mice compared to SAS-shCtrl-Luc-injected mice (Fig. 6D). Notably, mice with SAS/HRH1-KD tumors exhibited longer survival times than those with SAS/shCtrl tumors (Fig. 6E). Consistent with our in vitro findings, IHC staining of HRH1, ADAM9, and vimentin in tumor tissues from SAS-shHRH1-injected mice indicated downregulation of these proteins compared to control mice. In contrast, the epithelial marker E-cadherin was upregulated in tumor tissues from SAS-shHRH1-injected mice (Fig. 6F). Taken together, these results suggest that the ADAM9-mediated EMT may play a crucial role in HRH1-induced in vivo tumorigenicity and LN metastasis of OSCC.

### Treatment of HRH1 antagonists attenuates the HRH1-drived EMT and malignancy in OSCC cells

**Fig. 7:**
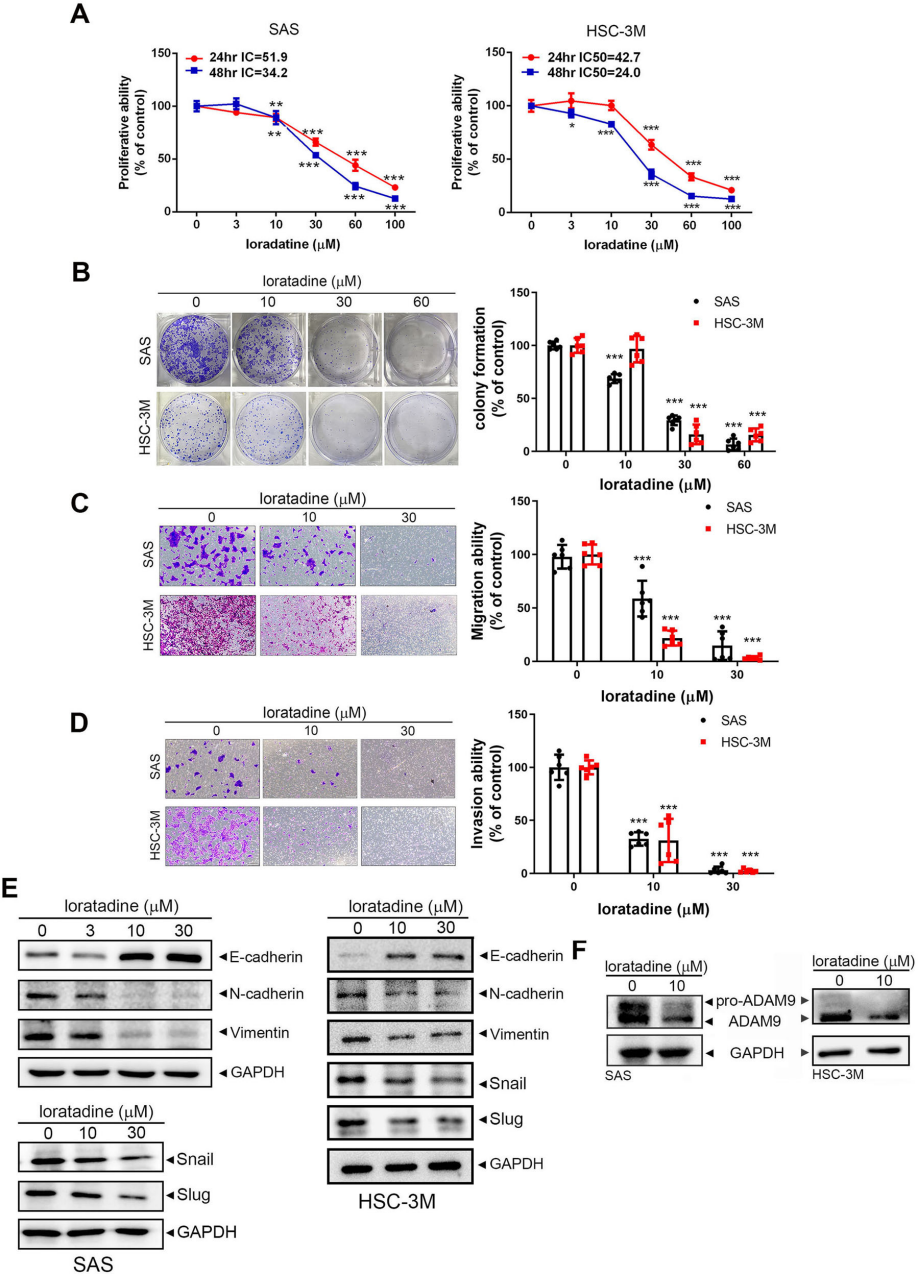
Loratadine treatment attenuates the HRH1-derived EMT and malignancy of OSCC cells. **A** SAS and HSC-3M cells were treated with various concentrations of loratadine for 24 and 48 h, and the half-maximal inhibitory concentration (IC_50_) of these cells was determined by an MTS assay. The colony-forming (**B**), migratory (**C**, and invasive (**D**) abilities of SAS and HSC-3M cells were determined after treatment with either vehicle or various concentrations of loratadine. The left panels display representative photomicrographs, while the right panels present values as the mean ± standard deviation (SD). **p < 0.01, ***p < 0.001 com*p*ared to the vehicle group. **E** Protein levels of E-cadherin, N-cadherin, vimentin, Snail, Slug, and ADAM9 in SAS and HSC-3M cells treated with loratadine for 24 h were assessed by Western blotting.

After delineating the oncogenic role and associated mechanisms of HRH1 in OSCC, our next step was to investigate the therapeutic potential of HRH1 inhibitors in OSCC. Treatment of SAS and HSC-3M cells with the second-generation HRH1-specific antagonist, loratadine, or its active metabolite, desloratadine, resulted in significant concentration-dependent inhibition of cell proliferation (Figs. 7A and S9A), colony formation (Figs. 7B and S9B), cell migration (Figs. 7C, and S9C) and invasion (Fig. 7D). Similar to HRH1-KD, loratadine and desloratadine demonstrated more-potent inhibitory effects on cell motility than on cell growth. Furthermore, loratadine treatment led to concentration-dependent upregulation of E-cadherin and downregulation of N-cadherin, vimentin, Snail, Slug, and ADAM9 in SAS and HSC-3M cells (Fig. 7E, F). Additionally, we observed that loratadine treatment in both OSCC cell lines reversed histamine-induced upregulation of Slug and ADAM9 (Fig. S10). Overall, these findings suggest that inhibition of HRH1 by a specific antagonist may represent a promising therapeutic strategy for impeding OSCC progression through blocking the ADAM9-mediated EMT process.

### Enhancing the therapeutic efficacy of HRH1 signaling blockade in OSCC can be synergistically achieved through targeting STAT3 signaling

**Fig. 8:**
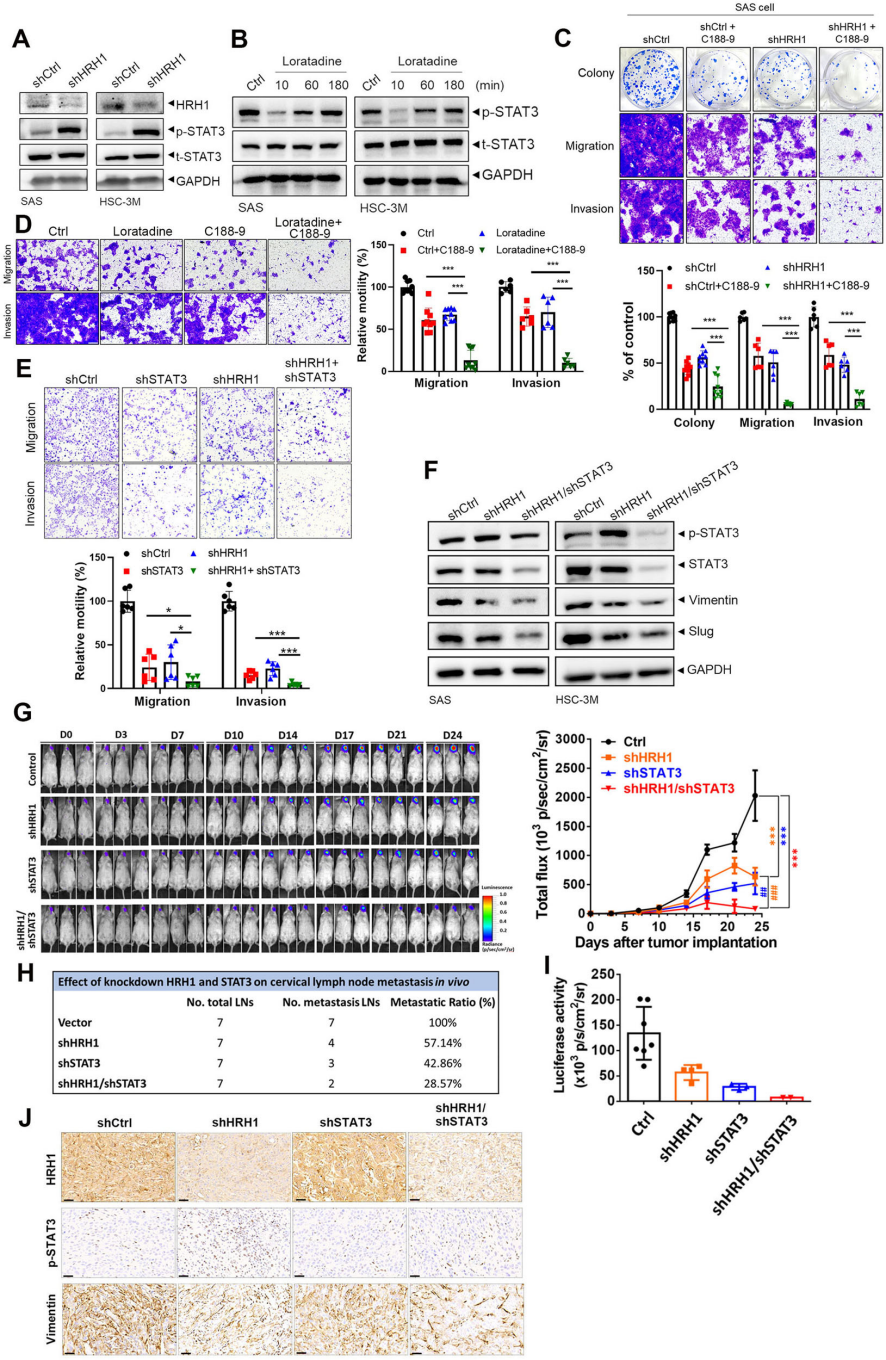
Synergistically enhancing the therapeutic efficacy of HRH1 signaling blockade in OSCC can be achieved by targeting STAT3 signaling. Protein levels of phosphorylated (p)-STAT3 and STAT3 in SAS and HSC-3M cells were assessed by Western blotting following HRH1-knockdown (**A**) or treatment with loratadine (10 µM) for the indicated time points (**B**). **C**‒**E** Different malignant properties, including colony-formation, migration, and invasion abilities, were analyzed in SAS or HSC-3M cells with HRH1 or STAT3 inhibition alone or combined inhibition of both targets using chemical inhibitors (10 µM loratadine and 10 µM C188-9) or shRNAs (shHRH1 and shSTAT3). **C**: shHRH1 + C188-9, **D**: loratadine+C188-9, **E**: shHRH1+shSTAT3. Data are shown as the mean ± standard deviation (SD), *n* = 2–3, two-tailed Student’s *t*-test. *p < 0.05, ***p < 0.001 com*p*ared to HRH1 or STAT3 inhibition alone. **F** Protein levels of p-STAT3, STAT3, and EMT-related proteins in SAS and HSC-3M cells were assessed by Western blotting following HRH1-knockdown or combined knockdown of HRH1 and STAT3. **G** NOD/SCID mice were orthotopically implanted with luciferase-tagged HSC-3M-expressing shCtrl, shHRH1, shSTAT3, or shHRH1+shSTAT3, and luciferase activity was detected twice per week with an IVIS (left panel). Quantitative analysis of the Xenogen imaging signal intensity (photons/s/cm^2^/sr) is shown in the right panel. Frequencies (**H**) and mean intensities of signals (**I**) of cervical lymph node (LN) metastasis by bioluminescent detection at the end of the study. **J** HSC-3M xenografts with different genetic manipulations described above were isolated to detect expressions of HRH1, p-STAT3, and vimentin by IHC staining. Original magnification, 400 × Scale bar, 30 µm.

In addition to TGF-β signaling and the EMT, GSEA results presented in Fig. 3B, E also indicate that STAT3 may be another downstream target of HRH1. Interestingly, the interleukin (IL)-6/Janus kinase (JAK)/STAT3 pathway was reported to contribute to tumor formation and progression in HNSCC by inducing the EMT [33]. To our surprise, we observed a significant elevation in STAT3 phosphorylation at Tyr-705 in SAS and HSC-3M cells with stable HRH1-KD (Fig. 8A). Treatment of both cell lines with loratadine or desloratadine initially suppressed STAT3 phosphorylation at early time points (10 min), but inhibition of STAT3 phosphorylation had recovered by 3 to 6 h after loratadine or desloratadine treatment (Figs. 8B and S11). These results suggest that activated STAT3 may function as a compensatory pathway for long-term HRH1 signaling blockade in OSCC cells. Herein, we employed various strategies to assess the therapeutic potential of combined targeting of HRH1 and STAT3 on OSCC progression. We discovered that inhibition of the STAT3 pathway using a specific inhibitor (C188-9) attenuated the growth and motility of SAS and HSC-3M cells and significantly enhanced the inhibitory effects induced by HRH1-KD (Figs. 8C and S12) as well as loratadine (Figs. 8D and S13) and desloratadine (Fig. S14) treatments, on OSCC growth or motility. Similar results were also observed in OSCC cells with double KD of STAT3 and HRH1 (Fig. 8E). Moreover, inhibition of STAT3 by C188-9 or shSTAT3 both attenuated HRH1-KD-induced STAT3 phosphorylation and enhanced the HRH1-KD-induced downregulation of vimentin, Snail, and Slug (Figs. 8F and S15). In the HSC-3M-Luc orthotopic graft mice model, photon emission detection revealed that the tumorigenic ability was suppressed in both HRH1-KD and STAT3-KD HSC-3M cells compared to the control group. Double KD of HRH1 and STAT3 in HSC-3M cells exhibited the strongest inhibitory effect on tumor growth compared to either HRH1-KD or STAT3-KD alone (Fig. 8G). Consistently, decreased frequencies (Fig. 8H) and intensities (Fig. 8I) of cervical LN metastasis in HRH1-KD HSC-3M mice were also enhanced under STAT3-KD. Results of the IHC analysis revealed upregulation of STAT3 phosphorylation in tumor tissues in which HRH1 was knocked down (Fig. 8J). IHC staining of vimentin in tumor tissues from HSC-3M/shSTAT3+shHRH1-injected mice showed the lowest expression compared to tumor tissues from either HSC-3M/shHRH1- or HSC-3M/shSTAT3-injected mice (Fig. 8J). Taken together, these results revealed that the combined targeting of HRH1 and STAT3 may offer a novel strategy for preventing EMT-mediated OSCC progression.

## Discussion

Currently, LN metastasis and therapeutic resistance remain major challenges in OSCC treatment [34], highlighting the urgent need for new therapeutic strategies. High HRH1 expression levels were recently reported in different cancers including HCC, colorectal cancer (CRC), breast cancer (BC), melanoma, and non-small cell lung cancer (NSCLC) [13, 35] and were correlated with growth, metastasis, or therapeutic resistance of these cancer types [17, 19, 36–38]. In this study, we found that HRH1 expression was frequently elevated in HNSCC and OSCC tissues and was associated with LN metastasis. The overexpression of HRH1 may serve as an independent prognostic marker for HNSCC patients. Additionally, genetic or pharmacological inhibition of HRH1 significantly impeded growth and metastasis in OSCC cell lines and mouse models. Several human trials showed that HRH1 antagonists (loratadine and desloratadine) can reduce risk and improve OS in patients with HCC [18], melanoma [39], and BC [40]. These results collectively indicate that HRH1 may be a novel therapeutic target for HNSCC treatment. However, the clinical impacts of HRH1 antagonists on HNSCC/OSCC are unknown and warrant further investigation.

In addition to HRH1, high concentrations of histamine were observed in various human malignancies such as CRC [41], BC [14], and melanoma [16]. Histamine was implicated in numerous tumor cell activities during tumor progression, including growth, invasion, and metastasis [18, 26], indicating its role as an autocrine growth factor that promotes cancer progression. In our study, we observed that the histamine concentration in the culture medium of OSCC cells was around 4 nM (Fig. S16). However, we found that only a high concentration of histamine (1 µM) could stimulate the colony-forming, migratory, and invasive abilities of OSCC cells. Histamine-induced cell motility was blocked when HRH1 was knocked down, suggesting that histamine promotes the growth and metastasis of OSCC cells by acting on HRH1. In the absence of high histamine concentrations, manipulating HRH1 expression still had a significant effect on OSCC cell behaviors, suggesting the existence of histamine-independent regulatory signals that activate HRH1 through interactions with unknown proteins or ligands. Both the histamine-dependent and—independent roles of HRH1 in cancer progression were also observed in HCC [17]. Potential molecules that interact with HRH1 and facilitate OSCC progression should be further investigated in the future.

The GSEA of RNA-Seq data from both TCGA HNSCC patients and HRH1-depleted OSCC cells revealed that TGF-β signaling and the EMT pathway were upregulated in the HRH1-enriched group. TGF-β signal activation was shown to induce the EMT process in HNSCC [27]. We validated that expressions of activated TGF-β and EMT-related markers, such as N-cadherin, vimentin, Snail, and Slug, were suppressed by pharmacological or genetic HRH1 inhibition. The inhibition of cell invasion caused by HRH1 depletion was significantly reversed when Snail or Slug was overexpressed in OSCC cells. Taken together, these data indicate that HRH1 modulates OSCC progression through the TGF-β-Snail/Slug-mediated EMT. Additionally, we surprisingly found that downregulation of the HRH1 protein and mRNA caused by HRH1-KD was also reversed by overexpressing Snail or Slug in OSCC cells, suggesting that the TGF-β‒Snail/Slug signal induced by HRH1 may exhibit positive feedback regulation on HRH1 in OSCC cells. According to a JASPAR database analysis, the HRH1 promoter region contains Snail/Slug-binding sites (Table S3). We propose that the TGF-β‒Snail/Slug signaling pathway might directly regulate HRH1 expression in OSCC cells. A previous study indicated that pharmacological inhibition of HRH1 can sensitize chemoresistant cells to epirubicin or adriamycin treatment by inhibiting the EMT and cellular efflux function of P-gp [37]. Therefore, it would be interesting to further evaluate the role of HRH1 in the chemosensitivity of OSCC cells.

In addition to promoting the EMT, HRH1 was observed to enhance migration and invasion by inducing MMP-2 upregulation in HCC cells [17]. Actually, TGF-β and MMPs mutually interact in inducing the EMT [30]. TGF-β activation involves the proteolytic cleavage of latency-associated peptide (LAP) by MMPs [42]. Our protease array results showed that ADAM9 was dramatically downregulated in HRH1-KD SAS cells. This downregulation was further validated by pharmacological or genetic HRH1 inhibition in OSCC cell lines and mouse models. Clinically, significant correlations between HRH1 and ADAM9 mRNA and protein levels were observed in HNSCC and OSCC specimens, respectively. Notably, ADAM9 overexpression significantly reversed inhibition of the EMT and cell motility induced by HRH1-KD, indicating that ADAM9 plays a crucial role in the HRH1-promoted EMT and OSCC progression. Moreover, ADAM9 was reported to cleave LAP to produce activated TGF-β in Th17 cells [43], suggesting that HRH1-mediated ADAM9 upregulation may promote the production of activated TGF-β and subsequently trigger the EMT process. It was demonstrated that HRH1 stimulates phospholipase to generate IP and DAG, leading to activation of PKC [44]. NF-κB is another signaling pathway activated by HRH1 [32]. Using PKC- and NF-κB-specific inhibitors, we demonstrated that PKC and NF-κB activation is involved in HRH1-mediated ADAM9 expression. Consistent with our findings, PKC activation was reported to induce ADAM9 upregulation in NSCLC cells [45], and NF-κB signaling was shown to promote ADAM9 expression in pancreatic cancer [46]. Given that other signaling pathways, such as extracellular signal-regulated kinase (Erk)1/2 activation, have been observed in HRH1 inhibition in BC cells [19] and shown to decrease ADAM9 expression in glioma cells [47], we cannot rule out the possibility that the ERK pathway may be involved in HRH1-mediated ADAM9 expression in OSCC cells, but this theory requires further investigation in the future.

The IL-6-JAK-STAT3-Snail signaling pathway was reported to induce EMT-mediated metastasis in OSCC [33]. Additionally, growing evidence suggests that STAT3 plays a crucial role in promoting cell proliferation, anti-apoptosis, angiogenesis, radiotherapy resistance, immune escape, and stem cell self-renewal, making it a potential molecular target and biomarker for OSCC [48]. In this study, we observed increased STAT3 activation (elevated phosphorylation level of STAT3; p-STAT3) in OSCC cells and mouse models following long-term suppression of HRH1 through pharmacological or genetic inhibition, suggesting that activated STAT3 may act as a compensatory pathway. Furthermore, combined pharmacological or genetic inhibition of HRH1 and STAT3 showed that STAT3 inhibition alone suppresses the EMT, growth, and metastasis of OSCC and significantly enhances the inhibitory effects of HRH1 inhibition in both in vitro and in vivo OSCC models. Regarding the roles of HRH1 and STAT3 in immune escape, HRH1 was reported to shift macrophages toward an M2-like tumor-associated macrophage (TAM) phenotype, with increased expression of the VISTA immune checkpoint, rendering CD8+ T cells dysfunctional [20]. HRH1 blockade was shown to upregulate major histocompatibility complex (MHC)-I expression in pancreatic cancer and enhance CD8^+^ T cell infiltration [49]. Additionally, STAT3 was shown to induce vascular endothelial growth factor (VEGF) secretion in larynx carcinoma cells, which subsequently enhances programmed death ligand 1 (PD-L1) expression in M2-like TAMs [50]. Therefore, the potential of combined targeting of HRH1 and STAT3, along with anti-PD-1 antibody therapy, warrants further investigation for treating OSCC.

**Fig. 9:**
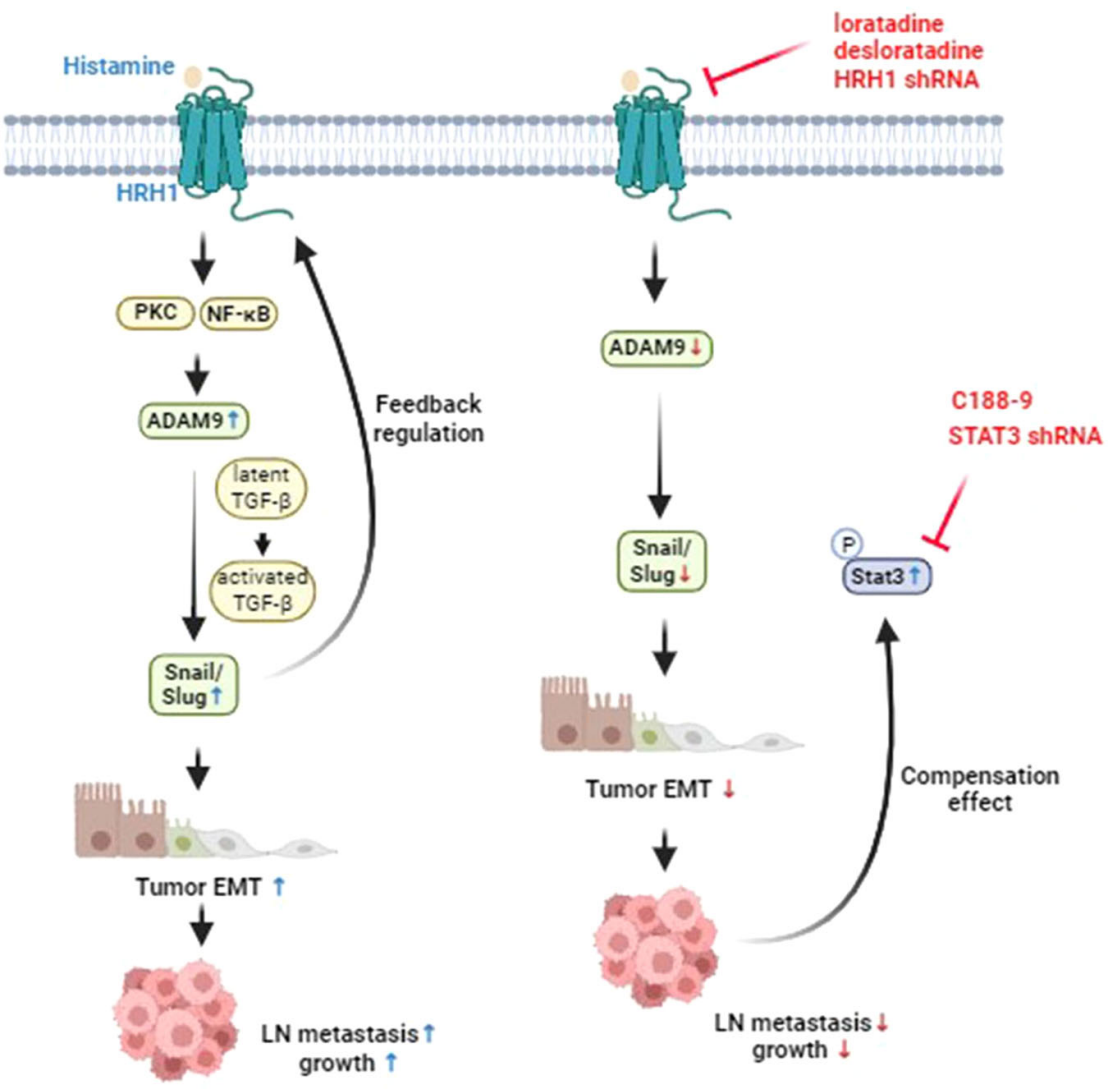
Schematic diagram illustrates the impact of HRH1 on the growth and lymph node (LN) metastasis of OSCC cells, along with their underlying mechanisms (created with BioRender.com). Left panel: HRH1 activation initiates PKC and NF-κB activation, subsequently prompting ADAM9-mediated upregulation of activated TGF-β and the Snail family, thereby inducing EMT-mediated OSCC progression. Cyclical increases in the HRH1-ADAM9-Snail/Slug axis contribute to the aggravation of EMT and OSCC growth and LN metastasis. Right panel: Activated STAT3 might act as a compensatory pathway in response to HRH1 signaling blockade in OSCC cells. A combined approach targeting both HRH1 and STAT3 using inhibitors or shRNAs presents a promising strategy for impeding EMT-mediated OSCC progression. PKC protein kinase C, NF-κB nuclear factor kappa B.

In summary, this study demonstrated that elevated HRH1 promotes the growth and LN metastasis of OSCC by inducing ADAM9-mediated upregulation of activated TGF-β, which in turn induces expressions of Snail family members and triggers EMT progression. We identified a novel cyclical mechanism involving the HRH1-ADAM9-Snail/Slug axis that drives OSCC progression. Additionally, we found that activated STAT3 acts as a compensatory pathway in response to HRH1 signaling blockade in OSCC cells. Therefore, a combined approach targeting both HRH1 and STAT3 presents a promising strategy for impeding EMT-mediated OSCC progression. The mechanisms deduced from our current study are schematically illustrated in Fig. 9.

## Materials and methods

### Materials

Histamine (H7125), C188-9 (573128), EVP4593 (SML0579), and dimethyl sulfoxide (DMSO) were sourced from Sigma-Aldrich (St. Louis, MO, USA). Loratadine (15625), desloratadine (16931), and chelerythrine (11314) were obtained from Cayman Chemical (Ann Arbor, MI, USA). Fetal bovine serum (FBS), antibiotics, molecular weight standards, trypsin-EDTA, and all medium additives were procured from Life Technologies (Gaithersburg, MD, USA). Antibodies against phosphorylated (p)-Akt (#9271), Akt (#9272), p-STAT3 (#9145), STAT3 (#9139), ADAM9 (#2099), E-cadherin (#3195), vimentin (#5741), N-cadherin (#13116), Snail (#3879), and Slug (#9585) were sourced from Cell Signaling Technology (Danvers, MA, USA). Antibodies targeting HRH1 (sc374621), transforming growth factor (TGF)-β (sc130348), and ADAM9 (sc377233) were purchased from Santa Cruz Biotechnology (Santa Cruz, CA, USA), while an antibody against GAPDH (60004-1-Ig) was obtained from Proteintech (Chicago, IL, USA).

### Data collection from bioinformatics analyses

To conduct a comprehensive analysis of HRH1 expression and its clinical importance in HNSCC patients, we employed several methodologies and datasets. Initially, we compared HRH1 expressions between tumor and normal tissues in HNSCC using two tools: TIMER 2.0 and TNMplot. These tools provided RNA sequencing (RNA-Seq) data from The Cancer Genome Atlas (TCGA) HNSCC patients (*N* = 520). Additionally, we utilized the GSE78060 dataset (*N* = 30) from the Gene Expression Omnibus (GEO) database, which contains data from advanced tongue squamous cell carcinoma patients. Within this dataset, we examined expression levels of HRH1 using two probes (205579_at and 205580_at), evaluating their differences between tumor and normal tissues, as well as exploring associations with LN metastasis and tumor sizes. For a survival analysis, we investigated HRH1 expression using TCGA and GSE31056 datasets. For TCGA HNSCC patients, Kaplan–Meier plots were generated to analyze overall survival (OS) and relapse-free survival (RFS). Cox regression analyses were employed to calculate hazard ratios (HRs), with the optimal cutoff for HRH1 expression determined based on minimizing *p* values. A multivariate Cox regression analysis, factoring in age, gender, pathological T and N stages, assessed HRH1 as an independent prognostic factor. The pathological M was excluded from the analysis due to a significant amount of missing data (198 patients lacked this information). In the GSE31056 dataset, a log-rank test evaluated the relationship between HRH1 expression and RFS. Moreover, a Pearson correlation analysis was performed on TCGA HNSCC data to assess gene expression correlations, with resulting coefficients and *p* values visualized in a correlation plot.

### Cell lines and cell culture

Human HNSCC cell lines, including several OSCC cell lines (CAL27, Ca9-22, FaDu, SAS, HSC-2, HSC-3, HSC-4, HSC-3M, SCC4, SCC9, SCC15, SCC25, and OECM1), were cultured in appropriate media, such as RPMI1640, Dulbecco’s modified Eagle medium (DMEM)/F12, DMEM, and minimum essential medium (MEM; Gibco, Grand Island, NY, USA). HOK and DOK keratinocyte cells were respectively cultured in oral keratinocyte medium (OKM, ScienCell Research Laboratories, Carlsbad, CA, USA) and DMEM (Gibco). All media (except OKM) were supplemented with 10% FBS, 100 units/mL penicillin, 100 μg/mL streptomycin, and 1% glutamine, and cells were maintained at 37 °C in a humidified 5% CO_2_ atmosphere.

### Western blot assay

Procedures for extracting total protein lysates and measuring protein concentrations were conducted as previously outlined [51]. Appropriate amounts of protein were then separated via sodium dodecyl sulfate polyacrylamide gel electrophoresis (SDS-PAGE) and transferred onto polyvinylidene difluoride (PVDF) membranes (Merck Millipore, Burlington, MA, USA). Subsequently, the membranes were incubated with specific primary antibodies followed by horseradish peroxidase-conjugated secondary antibodies. Afterward, the blots underwent washing with TBST buffer and were visualized using the ECL Western blotting reagent (Tools, New Taipei City, Taiwan), with chemiluminescence detected using the MultiGel-21 chemiluminescence imaging system (Top Bio, New Taipei City, Taiwan).

### Establishment of gene KD and overexpression of OSCC cells

The pLX304-HRH1 and pReceiver-M14-ADAM9 constructs were obtained from GeneCopoeia (Rockville, MD, USA), while short hairpin (sh)RNAs targeting HRH1, ADAM9, and STAT3 were acquired from the RNA Technology Platform and Gene Manipulation Core Facility at Academia Sinica (Taipei, Taiwan). pLEX-Snail and pCIneo-Slug constructs were obtained from Dr. T.C. Kuo (National Taiwan University, Taipei, Taiwan). Lentiviral particles expressing the specified shRNAs or pLX304-HRH1 were generated to knock down or overexpress the respective genes in OSCC cells for 24 h, following protocols outlined in our previous study [52]. For ADAM9, Snail, and Slug overexpression in OSCC cells, the Lipofectamine 3000 Transfection Reagent (Invitrogen, Carlsbad, CA, USA) was used, following procedures described in our prior work [52]. Targeting sequences of shRNAs were as follows: HRH1 shRNA-1, 5′-GCTCTGGTTCTATGCCAAGAT-3′; ADAM9 shRNA, 5′-GCCAGTATTATGATGCTCAAT-3′; and STAT3 shRNA, 5′-GCACAATCTACGAAGAATCAA-3′.

### Cell proliferation assay

HRH1-manipulated OSCC cells and their corresponding control cells were plated in 96-well plates at a density of 5 × 10^3^ cells/well, with each well containing 200 μl of complete culture medium. Following incubation for various time intervals (24 to 72 h), cell viability was assessed using a CellTiter 96 Aqueous One Solution Cell Proliferation Assay (MTS assay; Promega, Madison, WI, USA) as per the manufacturer’s guidelines. Data were obtained from three independent replicates.

### Plate colony-forming assay

SAS and HSC-3M OSCC cells expressing HRH1-V5, shNaa10p, or specific treatments were seeded in six-well plates at a density of 10^3^ cells/well and incubated for 24 h. Subsequently, the medium was refreshed every 2 days, and after 10 days of incubation, cells were fixed with methanol and stained with crystal violet. Colony counting was performed manually using ImageJ software (National Institutes of Health, Bethesda, MD, USA).

### Transwell migration/invasion assay

Cell motility assays, including migration and invasion assays, were conducted using 24-well transwells with an 8-μm pore size (Corning Costar, Corning, NY, USA), following previously described methods [53]. Briefly, for the migration assay, 8 × 10^4^ SAS cells or 6 × 10^4^ HSC-3M cells were seeded in the upper chamber without a coating. For the invasion assay, 10^5^ SAS cells or 8 × 10^4^ HSC-3M cells were plated in the upper chamber, which was coated with Matrigel (BD Biosciences, Bedford, MA, USA). Complete medium was added to the lower chamber as a chemoattractant. After incubation for 24 (HSC-3M) or 48 h (SAS), cells that had invaded the lower surface were fixed with methanol and stained with 0.5% crystal violet. The number of stained cells was counted in at least three random microscopic fields (×100 magnification).

### OSCC specimens

Fifteen paired OSCC tissues and adjacent non-tumor tissues were procured from Taipei Medical University Hospital, Wan-Fang Hospital, and Shuang Ho Hospital, Taiwan (TMU-JIRB no. N202204007). Furthermore, 177 OSCC specimens were acquired from Changhua Christian Hospital, Taiwan (IRB no. 220110), and assembled into a tissue microarray (TMA) by the Department of Pathology at Changhua Christian Hospital. Following construction, the TMA underwent staining with hematoxylin and eosin (H&E), and confirmation of morphologically representative lesions was conducted by two senior pathologists.

### Immunohistochemical (IHC) staining

HRH1 and ADAM9 expressions were assessed in a human OSCC TMA comprising 177 OSCC tissues through IHC staining, following established protocols [51]. Briefly, paraffin-embedded OSCC tissue sections were deparaffinized using xylene and rehydrated with a series of ethanol concentrations. Antigen retrieval was performed by boiling deparaffinized sections in 0.1 M citric acid buffer (pH 6.0), followed by overnight incubation at 4 °C with primary antibodies against ADAM9 (1:100) and HRH1 (1:200). Subsequently, slides were incubated with secondary antibodies at room temperature for 1 h, and signal development was conducted using a diaminobenzidine (DAB) kit (Boster, Wuhan, China). Images of stained TMAs were captured using a Motic Easy Scan Digital Slide Scanner (Motic, Schertz, TX, USA). The intensity of HRH1 and ADAM9 expression was evaluated by scoring the staining results under a light microscope, as previously described [51].

### Differentially expressed gene (DEG), pathway enrichment, and upstream regulator analysis

To delineate transcriptomic alterations mediated by HRH1 in HNSCC, we employed a gene set enrichment analysis (GSEA) on RNA-Seq data from both TCGA HNSCC patients and HRH1-depleted OSCC cells. For TCGA HNSCC cohort, patients were stratified into high HRH1 expression (top 5%) and low HRH1 expression (bottom 5%) groups, facilitating the GSEA. In RNA-Seq data of HRH1-depleted cells, genes with a false discovery rate (FDR) of <0.05 and fold change (FC) of >1.5 or <0.7 were respectively identified as potentially significantly upregulated or downregulated genes. Subsequently, the GSEA was conducted based on these FCs. We employed a weighted Kolmogorov-Smirnov test with 1000 permutations to calculate normalized enrichment scores and FDRs for each pathway. Pathways showing an FDR of <0.1 were considered significantly enriched, with analysis utilizing the Hallmark pathway database. Furthermore, we employed Qiagen’s Ingenuity Pathway Analysis (IPA) to investigate upstream regulators of HRH1-regulated genes. Significantly altered genes in HRH1-depleted cells were utilized to identify upstream regulators, encompassing transcription factors, kinases, and growth factors. *p* values were calculated based on the overlap between HRH1-regulated genes and known targets regulated by an upstream regulator. Activation z-scores were employed to predict activation states of upstream regulators, with positive and negative activation z-scores indicating activation or inhibition, respectively.

### Real-time reverse-transcription quantitative polymerase chain reaction (RT-qPCR)

Total RNA was isolated from OSCC cells using Trizol (ThermoFisher Scientific, Waltham, MA, USA) and then reverse-transcribed into complementary (c)DNA utilizing an iScript™ cDNA Synthesis Kit (Bio-Rad, Hercules, CA, USA). Subsequently, cDNA was subjected to an RT-qPCR using the TOOLS 2× SYBR qPCR Mix kit (Biotools, Taipei, Taiwan), with GAPDH serving as the internal control. Primer sequences utilized in the RT-qPCR were as follows: HRH1 forward: 5′-GCC GAG AGG ACA AGT GTG A-3′; HRH1 reverse: 5′-GGA GAC TCC TTC CCT GGT TT-3′; GAPDH forward: 5′-CTG GAG AAA CCT GCC AAG TAT GAT-3′; and GAPDH reverse: 5′-TTC TTA CTC CTT GGA GGC CAT GTA-3′.

### Protease array analysis

Protein lysates (200 μg) obtained from SAS/vector or SAS/shHRH1 cells were analyzed using a protease array kit containing 35 different protease capture antibodies printed in duplicate on nitrocellulose membranes (ARY021B, R&D Systems, Minneapolis, MN, USA). Following the manufacturer’s instructions, the pixel density of each spot on the developed x-ray film was quantified using Image-Pro Plus software. Spot densities were normalized to reference array spots and subsequently to controls.

### Coimmunoprecipitation (Co-IP)

To investigate the endogenous interaction between ADAM9 and HRH1, SAS cells were lysed in NETN lysis buffer (comprising 150 mM NaCl, 20 mM Tris-base, 0.5% NP-40, and 1 mM EDTA). Next, 2 mg of cell lysates was incubated with an anti-ADAM9 antibody overnight at 4 °C. Following this, lysates were subjected to incubation with 25 μl of immobilized protein A Sepharose beads for 1 h. Protein complexes were then washed five times with NETN buffer. Finally, proteins extracted from SAS cells were denatured in 5× sample dye and subsequently analyzed by Western blotting.

### In vivo OSCC orthotopic xenograft model

Six-week-old nonobese diabetic (NOD) SCID male mice were used in assays for tumor growth and cervical LN metastasis in an orthotopic graft model. All mice received the same anesthetic regimen using isoflurane Luciferase-tagged SAS or HSC-3M cells (5 × 10^5^) expressing shHRH1, shSTAT3, shHRH1/shSTAT3, or a control vector were resuspended in 20 μl of a 1:1 mixture of phosphate-buffered saline (PBS) and Matrigel and submucosally injected in the floor of the mouth using a 30-gauge needle. Tumor growth and LN metastasis from each group were monitored weekly using the Xenogen IVIS (in vivo imaging system) spectrum bioluminescence imaging (BLI) system (Caliper; Xenogen, Alameda, CA, USA). At the end of the experiment, primary tumors and cervical LNs were harvested, fixed, sectioned, and stained with the indicated antibodies. All animal studies were performed according to protocols approved by the Institutional Animal Care and Use Committee of Wan Fang Hospital (approval no.: WAN-LAC-111-018).

### Enzyme-linked immunosorbent assay (ELISA)

We seeded 5 × 10^5^ OSCC cells/well into 6 cm dishes for 48 h. Then, complete medium was replaced with serum-free medium for an additional 24 h. Supernatants were collected, and histamine concentrations were measured according to the manufacturer’s protocol using an LDN BAE-1000 histamine ELISA kit (LDN, Nordhorn, Germany).

### Statistical analysis

Statistical analyses of clinical data were described above. Values from both in vitro and in vivo studies are presented as the mean ± standard deviation (SD). Statistical analyses were conducted using SPSS vers. 20 (SPSS, Chicago, IL, USA), and quantified data were analyzed with GraphPad Prism 7 (GraphPad Software, San Diego, CA, USA). Differences between two groups were assessed using Student’s *t*-test.

## Supplementary information

Supplemental Material

Related Manuscript File

## Data Availability

All data generated or analyzed during this study are included in this published article and its additional files.
