# Supplementary material for: Cyclic increase in the histamine receptor H1-ADAM9-Snail/Slug axis as a potential therapeutic target for EMT-mediated progression of oral squamous cell carcinoma
Source: Cell Death Dis. 2025 Mar 20;16(1):191. doi: 10.1038/s41419-025-07507-1 (PMC11926216; doi:10.1038/s41419-025-07507-1)

**Supplemental Information**

**Title:**

**Cyclic increase in the histamine receptor H1-ADAM9-Snail/Slug axis as a potential therapeutic target for EMT-mediated progression of oral squamous cell carcinoma**

Yi-Fang Ding, Kuo-Hao Ho, Wei-Jiunn Lee, Li-Hsin Chen, Feng-Koo Hsieh, Min-Che Tung, Shu-Hui Lin, Michael Hsiao, Shun-Fa Yang, Yi-Chieh Yang, and Ming-Hsien Chien

Correspondence to: Dr. Yi-Chieh Yang (E-mail: ycyang@tmu.edu.tw) and Dr. Ming-Hsien Chien (E-mail: mhchien1976@gmail.com)

**Supplementary Table 1**.The gene candidates that were respectively up-regulated and down-regulated in HRH1 high (top 5%) comparing with HRH1 low (bottom 5%) patients .

| **Up-regulated genes in HRH1 high comparing with HRH1 low patients** | | | | |
| --- | --- | --- | --- | --- |
| *HRH1* | *ELK3* | *ITGB1* | *CDCP1* | *NT5E* |
| *KLF10* | *ACTN1* | *NEDD4* | *CA2* | *CAB39* |
| *MYO1B* | *LUZP1* | *MICALCL* | *TUBB6* | *CAV2* |
| *CDH3* | *SLC31A2* | *TGFBR2* | *SFXN3* | *BDKRB2* |
| *RAB31* | *TLDC1* | *KIFC3* | *CAV1* | *CLCA2* |
| *LIMA1* | *SLC16A1* | *SLC12A4* | *KCTD11* | *ZAK* |
| *PANX1* | *FMNL2* | *VCL* | *PXN* | *LAMA3* |
| *MSK18* | *PTPRE* | *LAMC2* | *MICAL2* | *ANO6* |
| *VDR* | *DOCK5* | *KLF7* | *MSN* | *TMEM92* |
| *MAP4* | *CAP1* | *BNC1* | *EHD2* | *FLNB* |
| **Down-regulated genes in HRH1 high comparing with HRH1 low patients** | | | | |
| *ABHD11* | *SPHK2* | *SMARCD2* | *C1orf35* | *CHTOP* |
| *ZSCAN16* | *RBBP8NL* | *TSSK6* | *FIZ1* | *D2HGDH* |
| *DUSP28* | *FLJ20216* | *WASH7P* | *STRADA* | *DYNLT2* |
| *ZNF48* | *ACP6* | *RPP25* | *ING5* | *MLLT6* |
| *LINC00938* | *TOR3A* | *MIR600HG* | *CRYGS* | *CCDC101* |
| *C6orf136* | *CNNM3* | *ZNF672* | *ZNF768* | *HAUS8* |
| *REPIN1* | *PAXIP1-AS1* | *ZNF692* | *ZNF789* | *CREB3L4* |
| *ATG4D* | *SNHG20* | *FBRSL1* | *SCARNA12* | *ANKMY1* |
| *FGFBP3* | *MRPL34* | *SUSD4* | *NARF* | *ATP6V0E2* |
| *USP21* | *C2orf68* | *FAM127C* | *SPATA41* | *TIGD3* |

**Supplementary Table 2.** TGF-β-activated gene candidates downregulated (n=127) and upregulated (n=31) in HRH1-depleted cell lines .

| **Down-regulated genes in shHRH1 group** | | | | |
| --- | --- | --- | --- | --- |
| *FCGR3B* | *CAV2* | *RNF152* | *JAG1* | *FGF2* |
| *C2* | *HSPB1* | *TENM4* | *XBP1* | *DUSP4* |
| *ENO3* | *FNDC3B* | *NBEA* | *S100A10* | *ITGAE* |
| *PDE4A* | *PDE7A* | *VIPR1* | *LTBP2* | *VCAN* |
| *SRM* | *MAP3K1* | *ACP3* | *LOXL2* | *MSN* |
| *TUBB2A* | *F2RL1* | *TP63* | *VEGFC* | *P2RY6* |
| *HSD17B10* | *LAMC1* | *ALPP* | *HTRA1* | *TCF7L1* |
| *PSMC3* | *ANXA8L1* | *KRT5* | *SERPINE2* | *KDELR3* |
| *NOP58* | *CHST11* | *GDF15* | *CSPG4* | *SOX4* |
| *PRPS1* | *PIM1* | *CFH* | *ACSS2* | *ID1* |
| *HSP90AA1* | *BMP2* | *HCAR2* | *FST* | *BGN* |
| *IL11* | *ASS1* | *NUPR1* | *IL1A* | *ABCA1* |
| *ADAM12* | *MBNL2* | *PDGFA* | *LTBP1* | *ABCG1* |
| *CDKN1A* | *CNN2* | *VIM* | *LAMC2* | *HSPG2* |
| *TGM2* | *MYO1C* | *SCD* | *H19* | *VCL* |
| *TGFBR1* | *COL4A2* | *INHBA* | *NOTCH1* | *NLRP3* |
| *DUSP1* | *PODXL* | *PTPRK* | *CCN5* | *ITGB6* |
| *FN1* | *ITGBL1* | *LINC00842* | *PLA2G4A* | *ITGB1* |
| *NPPB* | *BCL9* | *HES1* | *NCAM1* | *THBS2* |
| *RRAD* | *KRT14* | *INPP5D* | *LEF1* |  |
| *TUBA1A* | *SREBF2* | *ITGAV* | *FOS* |  |
| *CSF2* | *ACVR1* | *TWIST1* | *ACTA2* |  |
| *IL23A* | *RAB31* | *TIMP3* | *FERMT1* |  |
| *CCL20* | *ITPR2* | *ENPP1* | *MYLK* |  |
| *FAP* | *RHBDD1* | *F3* | *AHNAK* |  |
| *BDNF* | *DSC2* | *NR4A1* | *ITGA6* |  |
| *IL6* | *CCNG2* | *RUNX2* | *TSC22D3* |  |

**Supplementary table 2 continued.**

| **Upregulated genes in shHRH1 group** | | | |
| --- | --- | --- | --- |
| *RARG* | *INHBB* | *EREG* | *CDKN2B* |
| *CSF1* | *CCR1* | *FOSB* |  |
| *JUNB* | *BHLHE40* | *NEDD9* |  |
| *PTGS2* | *MRC2* | *SMAD6* |  |
| *PDCD4* | *MMP14* | *COL5A1* |  |
| *IGFBP7* | *ANKRD1* | *ZFP36* |  |
| *FBLN2* | *COL6A3* | *TXNIP* |  |
| *NOS3* | *MMP11* | *ITGA3* |  |
| *ITGB2* | *VEGFA* | *FOXO1* |  |
| *PSAT1* | *TGFBI* | *SMAD7* |  |

**Supplementary Table S3.** Predicted binding sites for Snail1 and Snail2 were identified on the promoter regions of histamine receptor H1 (HRH1), extending up to 2 kb upstream of the transcription start site

| Name | Relative score | Start | End | Strand | Sequence |
| --- | --- | --- | --- | --- | --- |
| SNAI2 | 0.9836904 | 849 | 857 | + | TACAGGTGC |
| SNAI2 | 0.9034771 | 1116 | 1124 | + | CCCAGGTGC |
| SNAI2 | 0.9007551 | 474 | 482 | + | CTCAGGTGA |
| SNAI2 | 0.884153 | 43 | 51 | + | AGCAAGTTG |
| SNAI2 | 0.8566865 | 1936 | 1944 | + | GACAAGTTA |
| SNAI1 | 0.9578267 | 849 | 858 | + | TACAGGTGCC |
| SNAI1 | 0.8995012 | 474 | 483 | + | CTCAGGTGAA |
| SNAI1 | 0.8789645 | 1116 | 1125 | + | CCCAGGTGCC |

Binding sites with a position weight matrix (PWM) score of >0.85 were considered significant.

**Supplementary Figure S1.** Data on the differentiation of histamine receptor H1 (HRH1) levels between tumor tissue and normal tissue in various cancers were obtained and adapted from the TIMER2.0 website (http://timer.cistrome.org/).

**
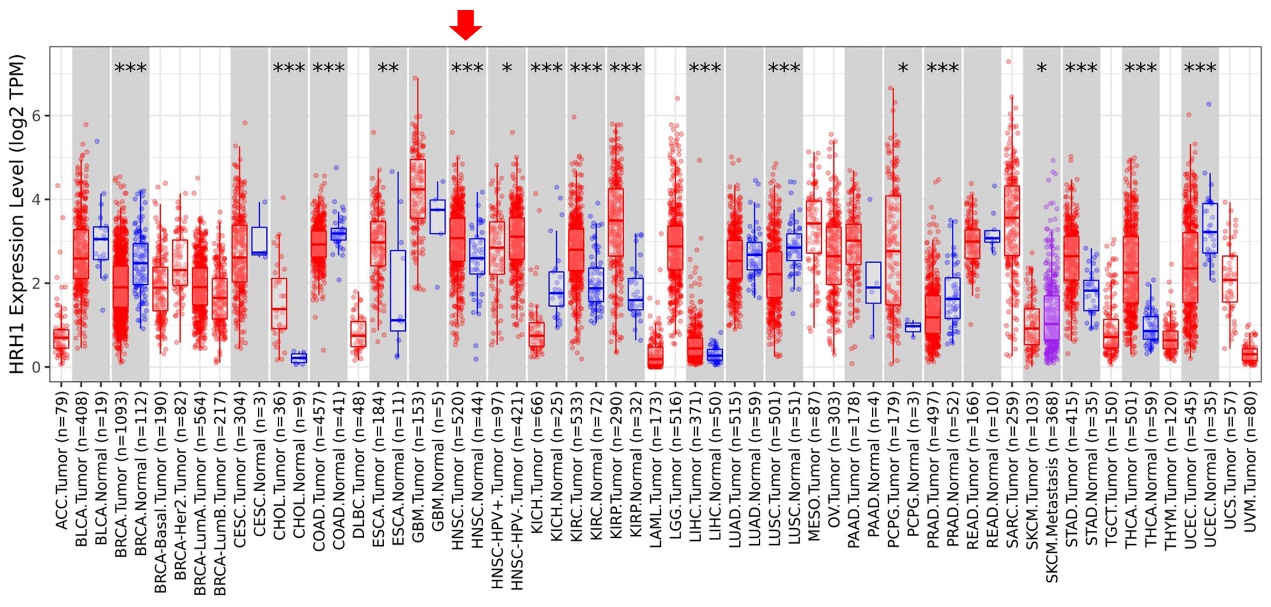
**

**Supplementary Figure S2.** (**A**) Expression levels of HRH1 in different T stages were determined through analysis of the GSE78060 OSCC cohort, using probes 205579_at and 205580_at. (**B**) Kaplan-Meier plot illustrating the relapse-free survival of patients with head and neck squamous cell carcinoma (HNSCC), categorized based on their HRH1 mRNA levels (high or low), was extracted from the GSE31056 dataset.


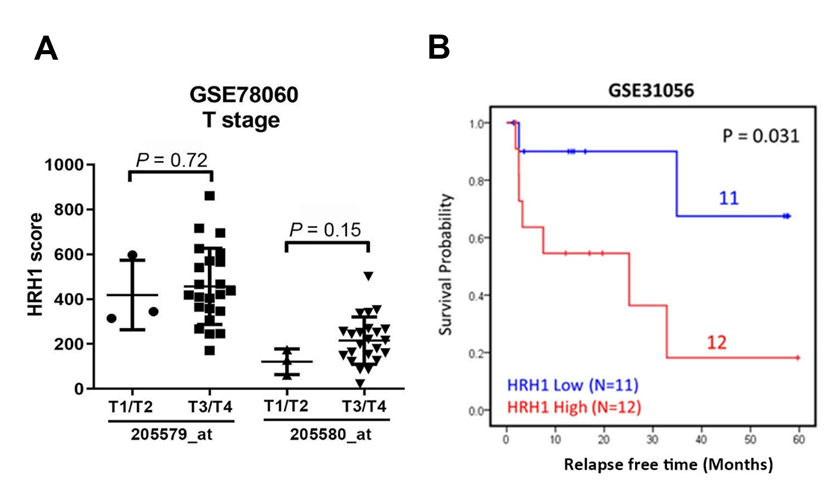


**Supplementary Figure S3.** (**A** and **B**) Impacts of histamine treatment (1 µM) on colony formation, migration, and invasion were respectively evaluated in SAS and HSC-3M cells through a colony-forming assay (**A**) and transwell migration/invasion assays (**B**). Data are shown as the mean ± standard deviation (SD). * *p*<0.05, *** *p*<0.001, compared to the vehicle control group.


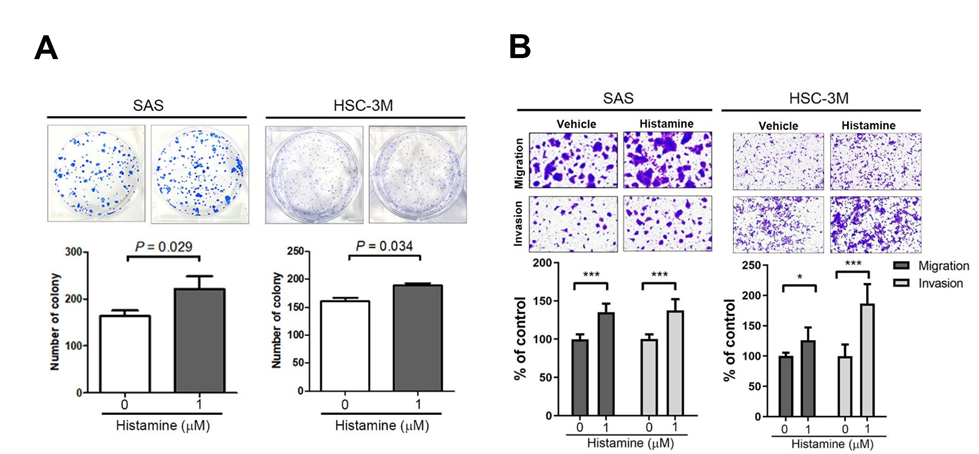


**Supplementary Figure S4.** Effects of histamine treatment (1 µM) on migration and invasion were assessed in SAS cells, with or without HRH1-knockdown, using transwell migration/invasion assays. Data are presented as the mean ± standard deviation (SD). * *p*<0.05, compared to the vehicle control group. ^###^ *p*<0.001, compared to the histamine-treated alone group. ^&^*p*<0.05, ^&&^*p*<0.01, compared to the histamine/shHRH1 group.


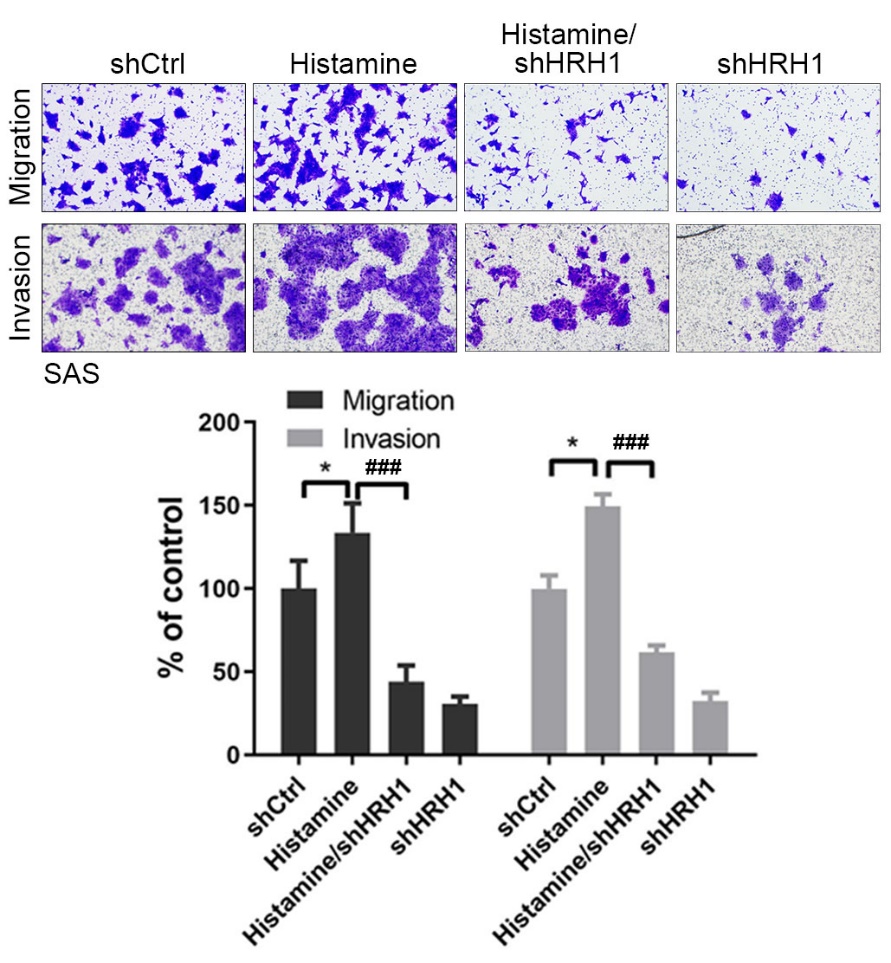

**Supplementary Figure S5.** Histamine receptor H1 (*HRH1*) mRNA expression was assessed via an RT-qPCR in SAS cells subjected to HRH1-knockdown (KD) or HRH1-KD combined with Snail or Slug overexpression. Results are displayed as the mean ± standard deviation (SD). * *p*<0.05, compared to the control group. ^#^ *p*<0.05, compared to the HRH1-KD alone group.


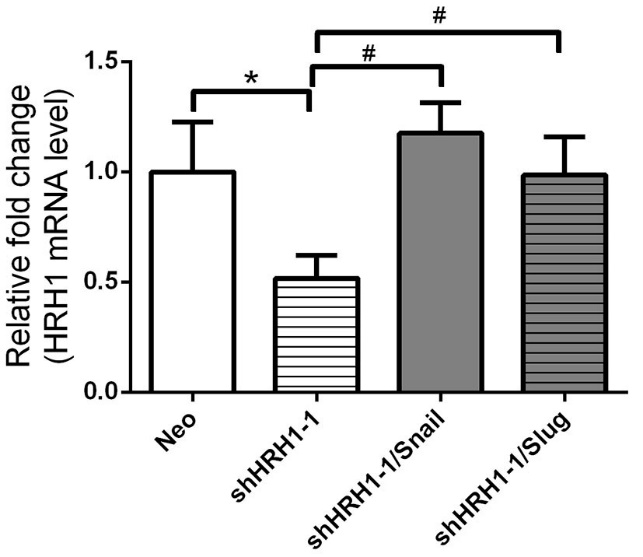


**Supplementary Figure S6.** (**A** and **B**) ADAM9 mRNA expression levels in paired adjacent and unpaired normal and tumor tissues, respectively derived from patients with head and neck squamous cell carcinoma (HNSCC) (**A**) and oral squamous cell carcinoma (OSCC) (**B**). Data for TCGA HNSCC were retrieved from TNMplot, while data for OSCC were obtained from the GSE78060 dataset. (**C**) Expression levels of ADAM9 mRNA in OSCC with different N (node) stages were determined through analysis of the GSE78060 dataset. For TCGA cohorts, the gene expression was normalized by count per millions (CPM). While, for GSE78060, the gene expression was normalized by MAS5.

**
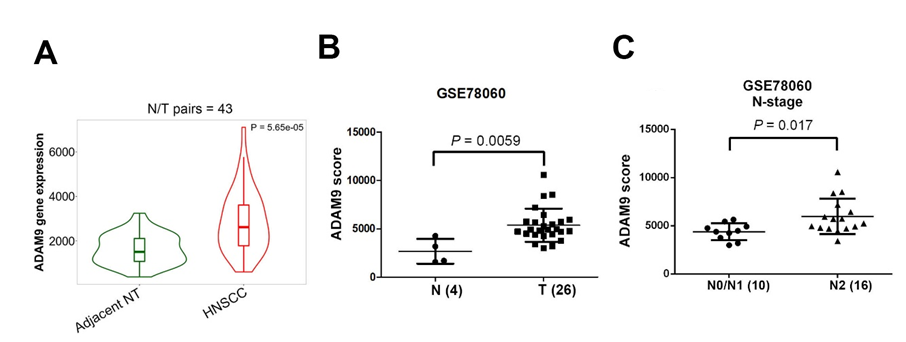
**

**Supplementary Figure S7.** (**A**) Impact of HRH1 overexpression on ADAM9 expression in HSC-3M cells was assessed via Western blotting (WB) using various ADAM9-specific antibodies. (**B**) Effects of histamine treatment for 24 h on HRH1 and ADAM9 expressions in SAS cells were evaluated by WB. (**C**) Coimmunoprecipitation (Co-IP) and WB revealed no binding between ADAM9 and HRH1 in SAS cells.

**
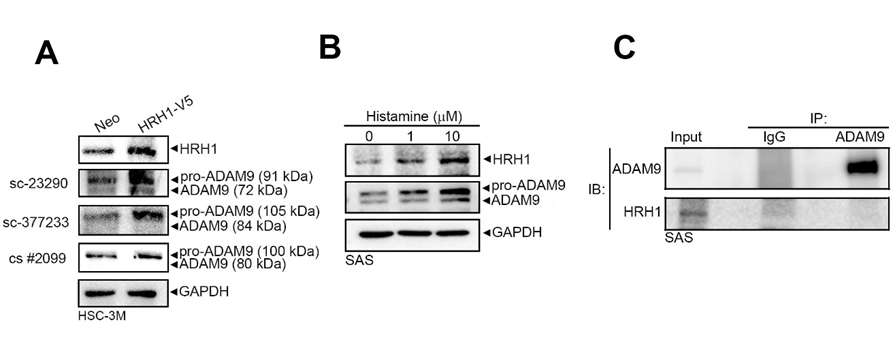
**

**Supplementary Figure S8.** Cell migratory ability was evaluated by a transwell migration assay in HSC-3M cells subjected to HRH1-knockdown (KD) or HRH1-KD combined with ADAM9 overexpression. Results are displayed as the mean ± standard deviation (SD). *** *p*<0.001, compared to the control group. ^#^ *p*<0.05, compared to the HRH1-KD alone group.

**
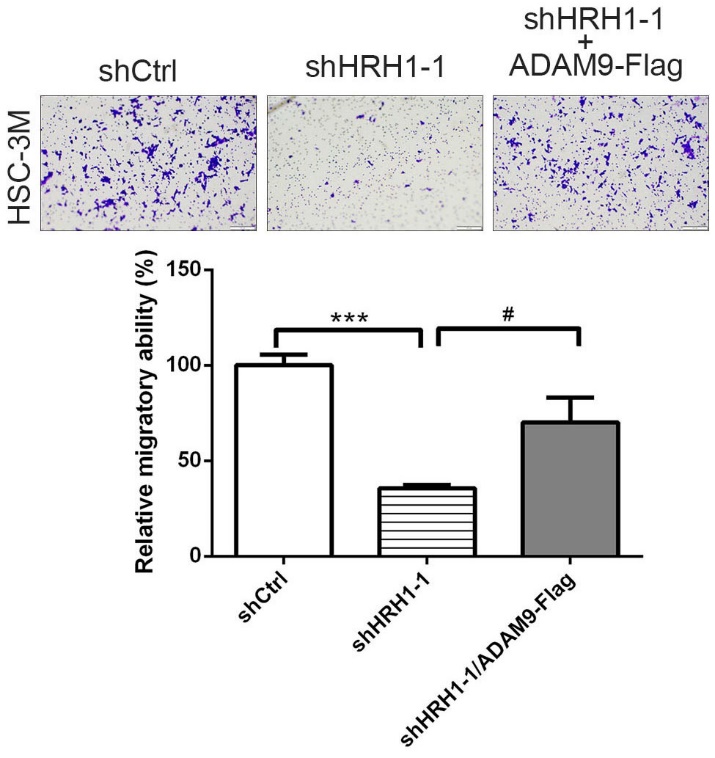
**

**Supplementary Figure S9.** (**A**-**C**) SAS or HSC-3M cells were treated with various concentrations of desloratadine for 24 or 48 h, and the half-maximal inhibitory concentration (IC_50_) on cell viability was determined by an MTS assay (**A**). Colony-forming (**B**) and migratory (**C**) abilities of cells were respectively determined by a colony-formation assay and transwell migration assay. Results are displayed as the mean ± standard deviation (SD). * *p*<0.05, ** *p*<0.01, *** *p*<0.001, compared to the control group.


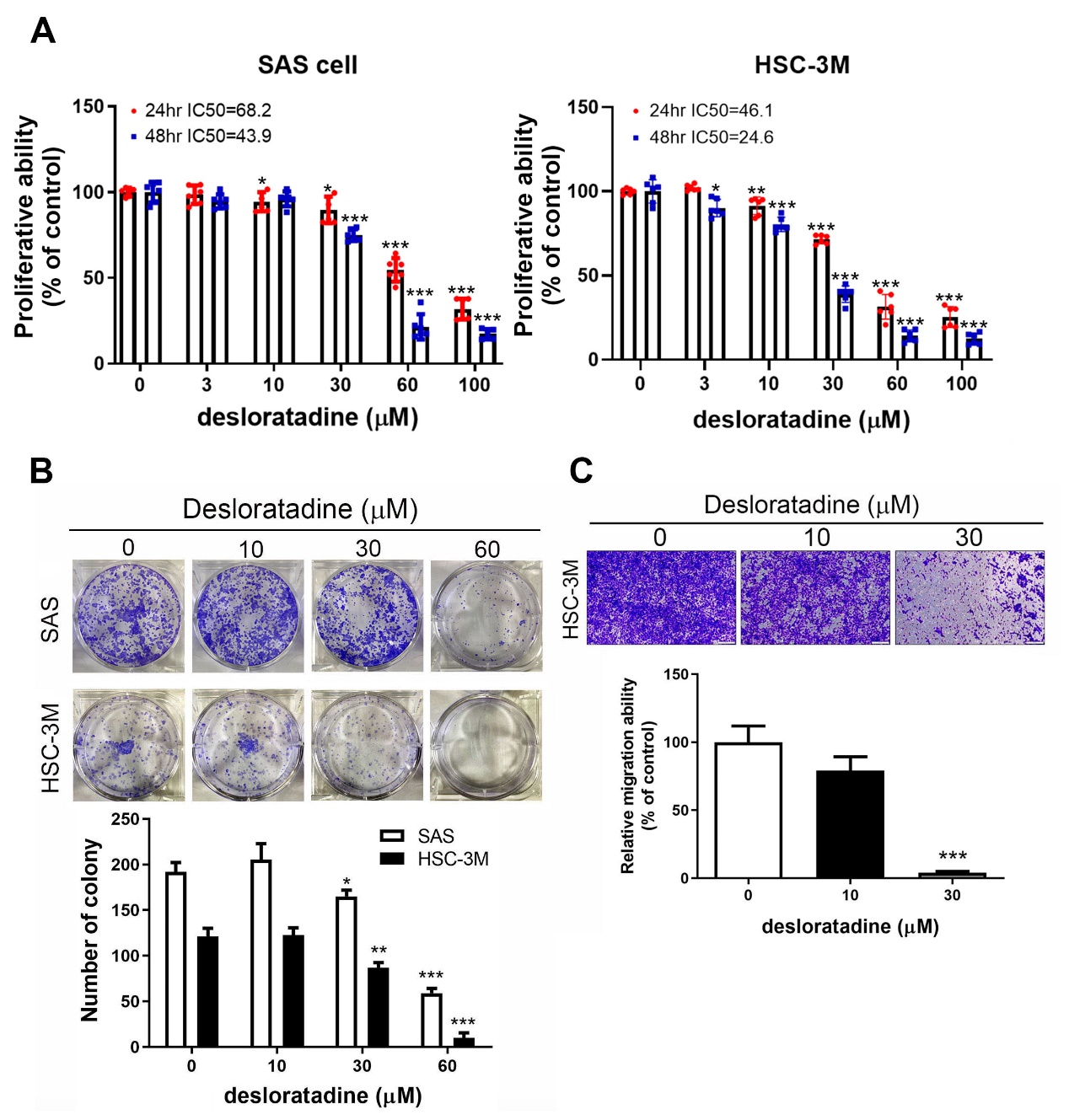


**Supplementary Figure S10.** Slug and ADAM9 protein levels were evaluated by Western blotting in SAS and HSC-3M cells treated with either 1 µM histamine or a combination of histamine and 10 µM loratadine.


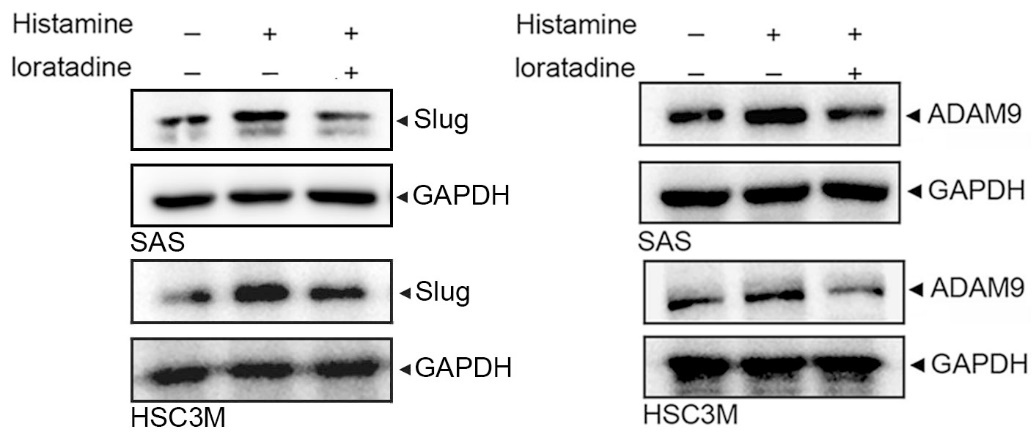


**Supplementary Figure S11.** Protein levels of phosphorylated (p)- STAT3 and STAT3 in SAS and HSC-3M cells were assessed by Western blotting following treatment with desloratadine (30 µM) for indicated time points.


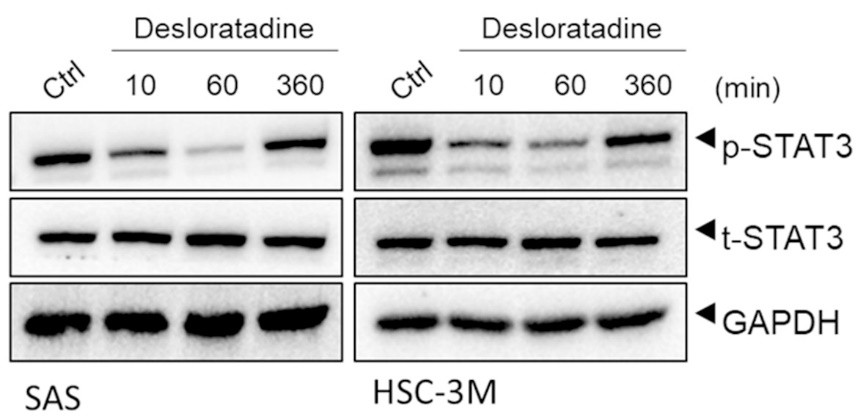


**Supplementary Figure S12.** Cell-migratory ability was evaluated by a transwell migration assay in HSC-3M cells subjected to HRH1-knockdown (KD), C188-9 treatment, or HRH1-KD combined with C188-9 treatment (*n*=3). Results are displayed as the mean ± standard deviation (SD). ** *p*<0.01, *** *p*<0.001, compared to the control group. ^##^ *p*<0.01, ^###^ *p*<0.001 compared to the HRH1-KD or C188-9 treatment alone group.


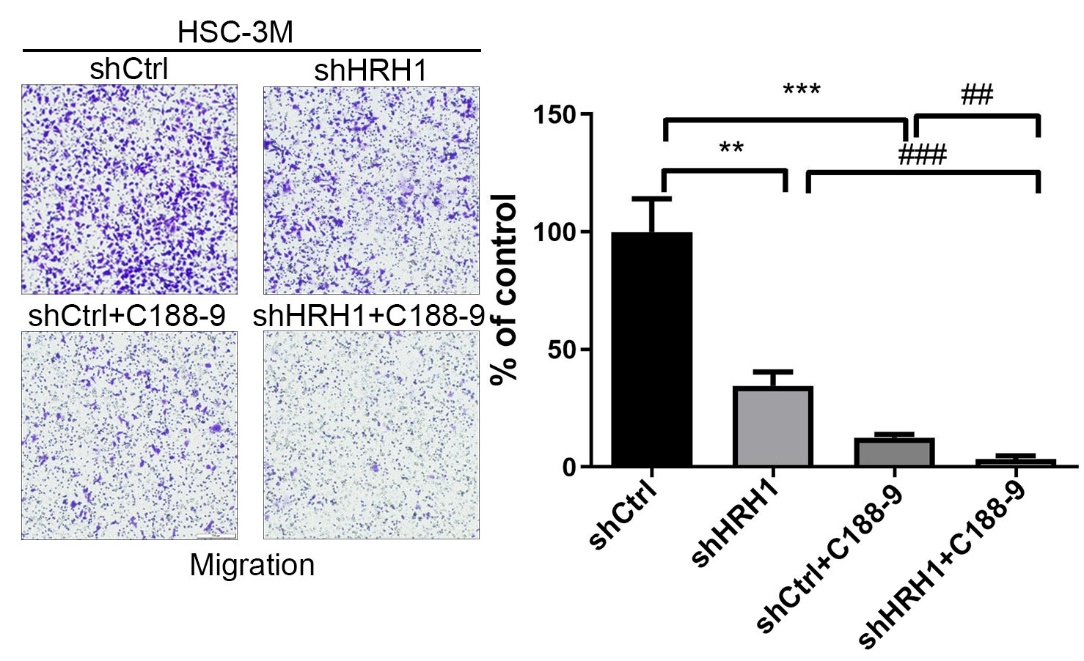


**Supplementary Figure S13.** Cell migratory and invasive abilities were respectively assessed using transwell migration and Matrigel invasion assays in HSC-3M cells treated with loratadine or C188-9 alone, or in combination. Results are displayed as the mean ± standard deviation (SD). * *p*<0.05, ** *p*<0.01, *** *p*<0.001, compared to the loratadine or C188-9 treatment alone group.

**
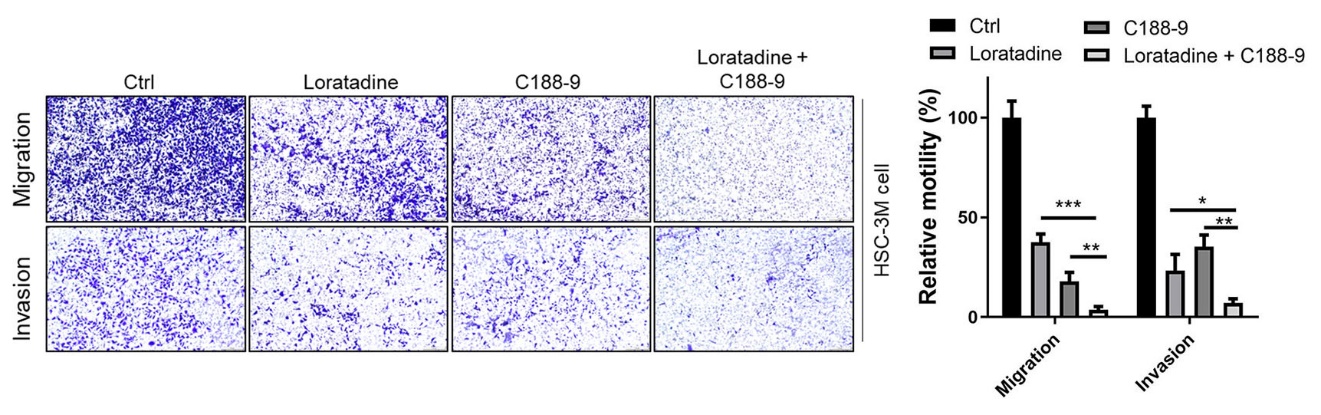
**

**Supplementary Figure S14.** Cell migratory and invasive abilities were respectively assessed using transwell migration and Matrigel invasion assays in HSC-3M and SAS cells treated with desloratadine (20 µM) or C188-9 (10 µM) alone, or in combination. Results are displayed as the mean ± standard deviation (SD). * *p*<0.05, ** *p*<0.01, *** *p*<0.001, compared to the desloratadine or C188-9 treatment alone group.

**
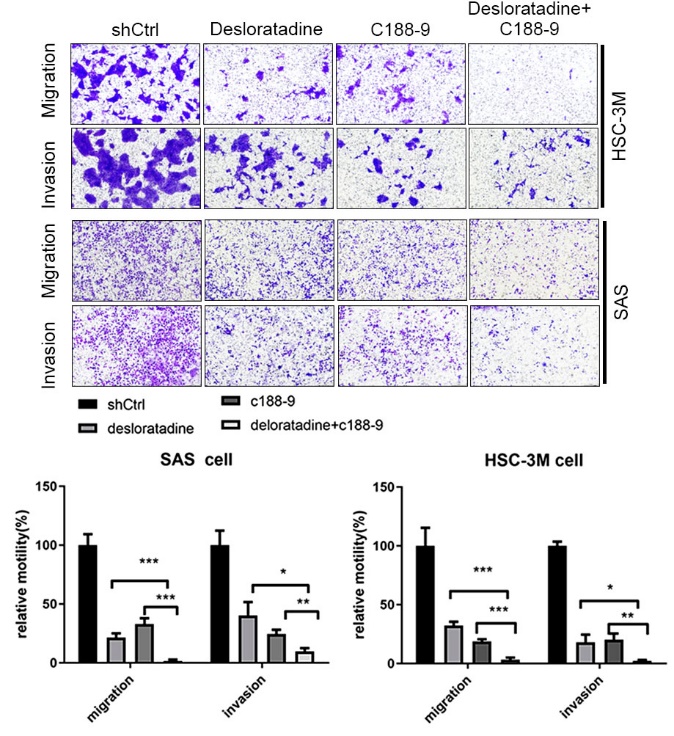
**

**Supplementary Figure S15.** Protein levels of HRH1, phosphorylated (p)- STAT3, vimentin, Slug, and Snail were assessed by Western blotting in SAS and HSC-3M cells subjected to HRH1-knockdown (KD), C188-9 treatment, or HRH1-KD combined with C188-9 treatment.


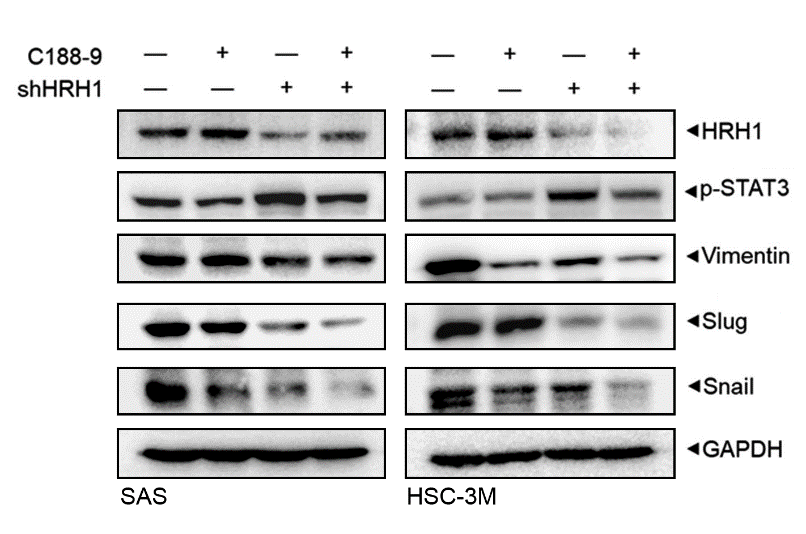


**Supplementary Figure S16.** Histamine levels in culture medium from the indicated OSCC cells were detected by an ELISA. Left panel, The standard curve of 0, 0.5, 1.5, 5, 15, and 50 ng/ml histamine and their corresponding OD values. Right panel, OD values and estimated histamine concentrations of OSCC cell lines.


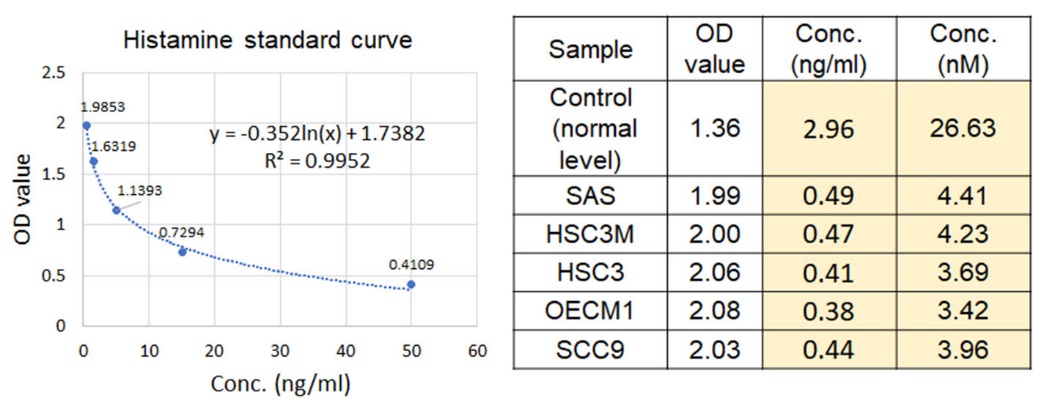

Supplement: Supplementary file 1 — Supplemental Material [file 41419_2025_7507_MOESM1_ESM.docx]
