# Supplementary material for: Cyclic increase in the histamine receptor H1-ADAM9-Snail/Slug axis as a potential therapeutic target for EMT-mediated progression of oral squamous cell carcinoma
Source: Cell Death Dis. 2025 Mar 20;16(1):191. doi: 10.1038/s41419-025-07507-1 (PMC11926216; doi:10.1038/s41419-025-07507-1)
Supplement: Supplementary file 2 — Related Manuscript File [file 41419_2025_7507_MOESM2_ESM.pdf]

Fig.2A

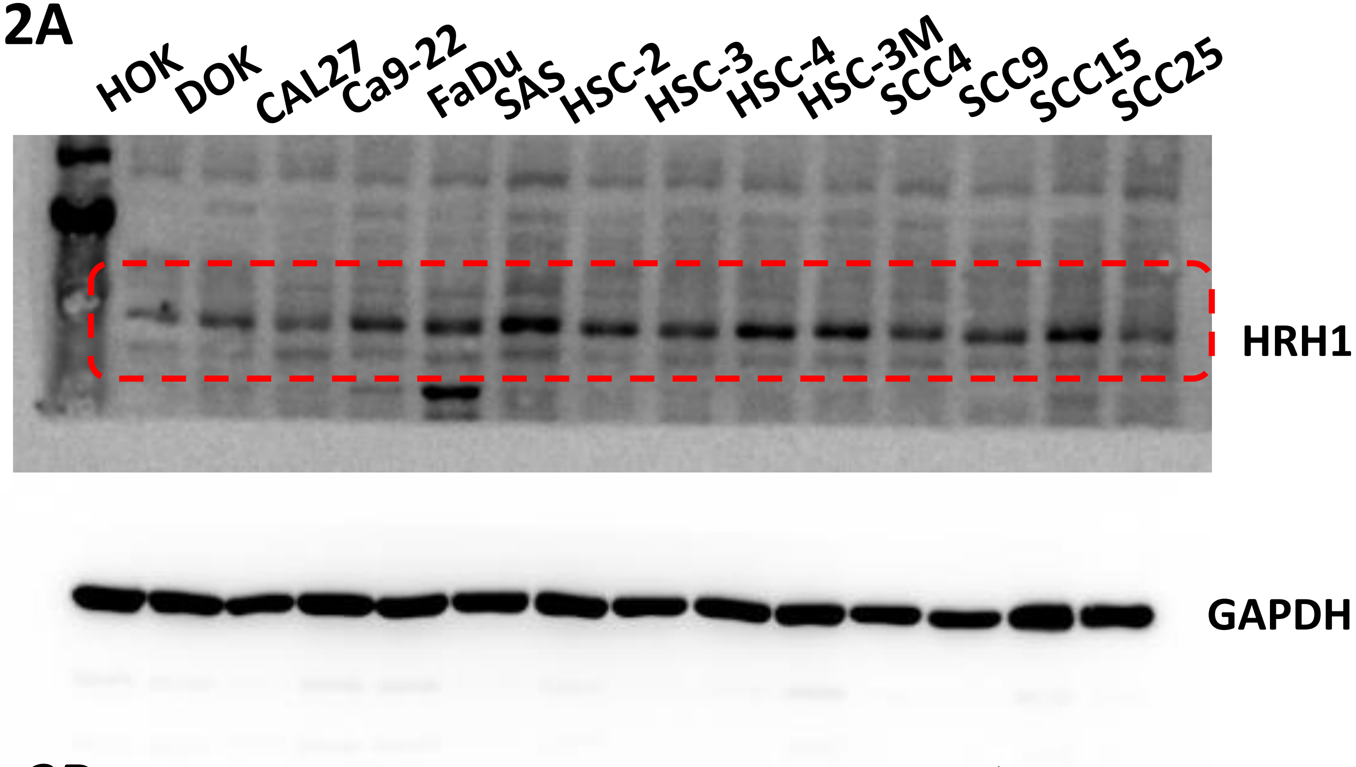


Fig.2F

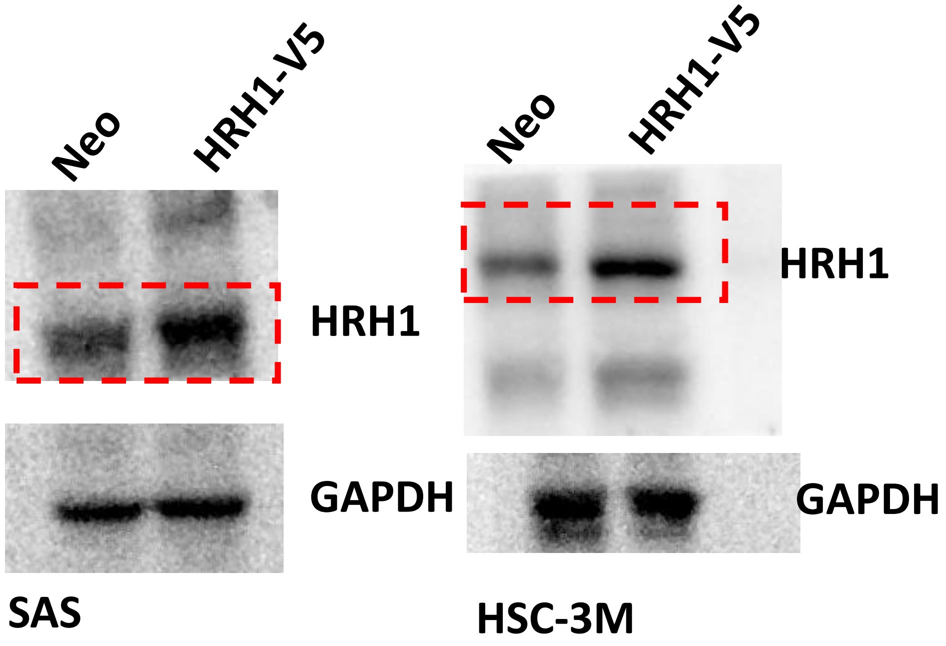


Fig.2B

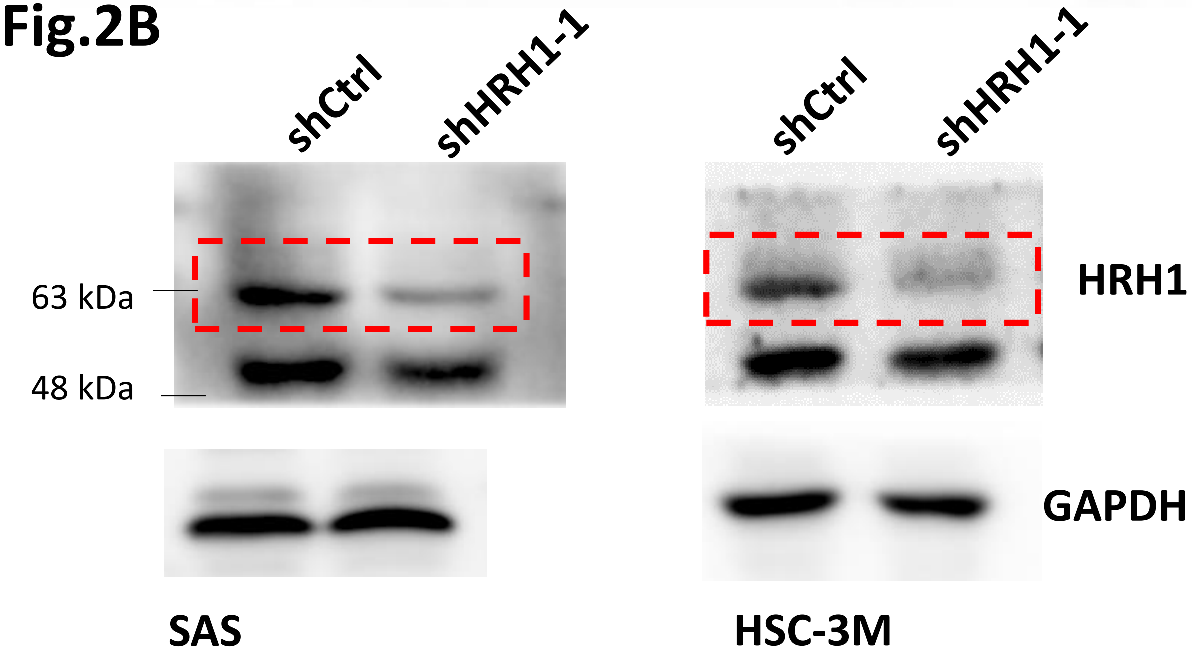

Fig.4A

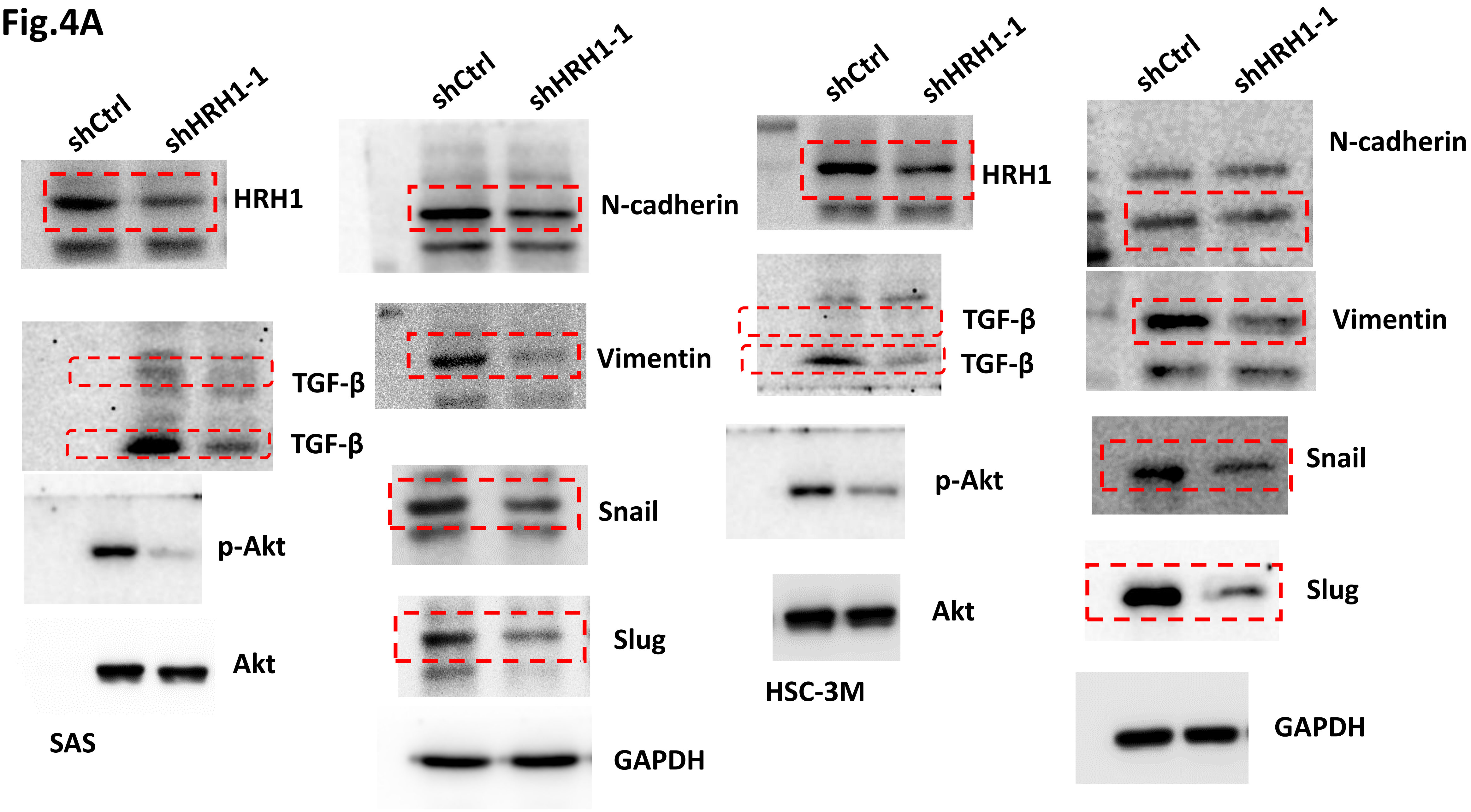
shCtrl
shHRH1-1
HRH1
TGF-β
TGF-β
p-Akt
Akt
SAS
N-cadherin
Vimentin
Snail
Slug
GAPDH
HSC-3M

Fig.4B

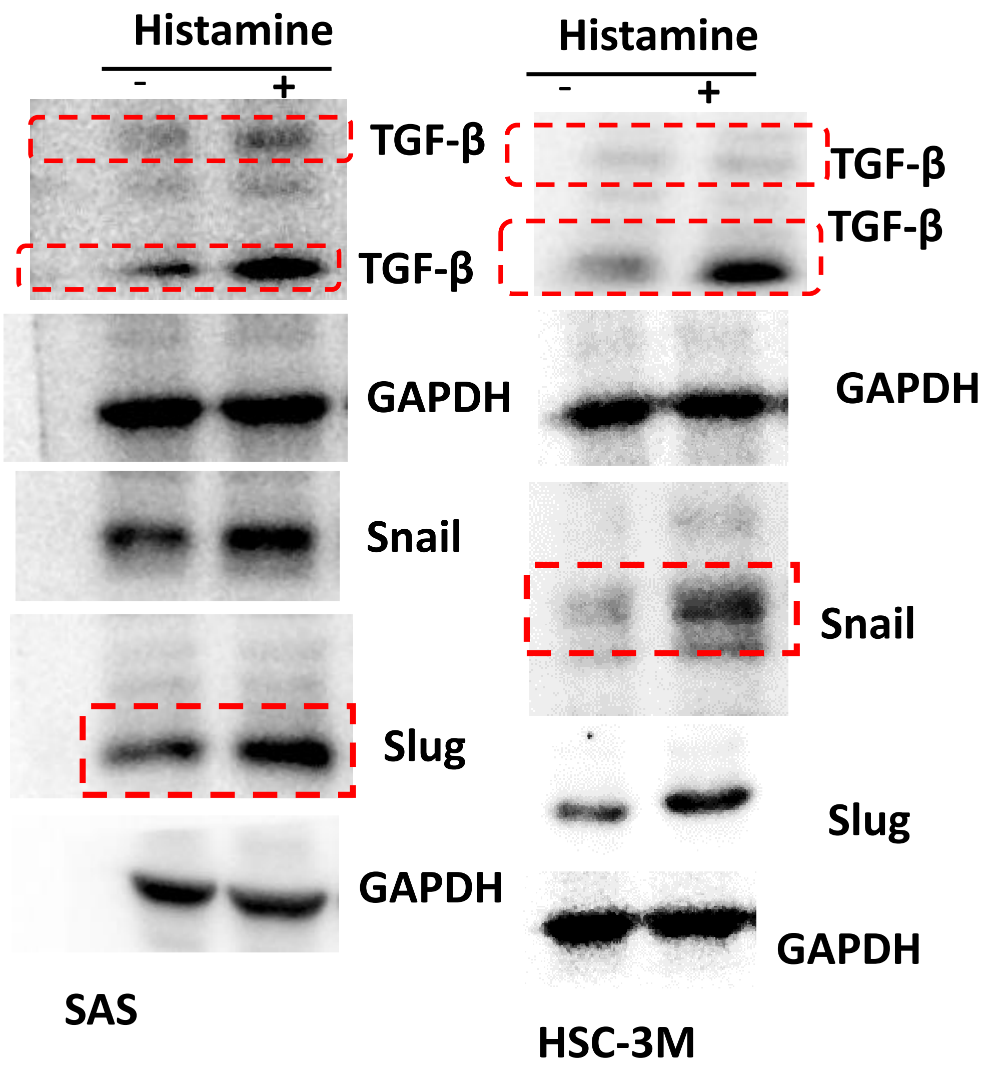
Histamine
-
+
TGF-β
TGF-β
GAPDH
Snail
Slug
GAPDH
SAS
Histamine
-
+
TGF-β
TGF-β
GAPDH
Snail
Slug
GAPDH
HSC-3M

Fig.4C

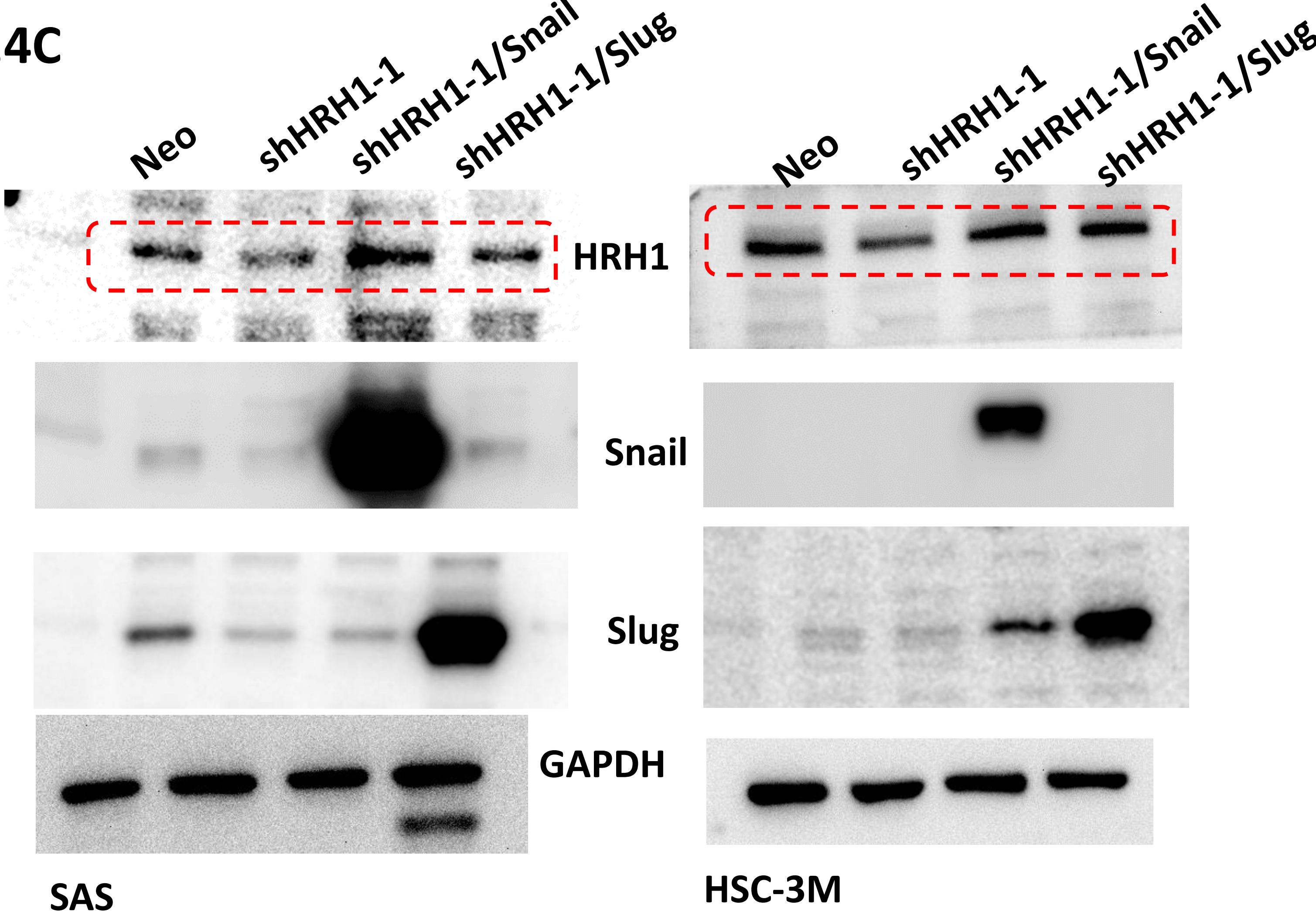
Neo
shHRH1-1
shHRH1-1/Snail
shHRH1-1/Slug
HRH1
Snail
Slug
GAPDH
SAS
Neo
shHRH1-1
shHRH1-1/Snail
shHRH1-1/Slug
HSC-3M

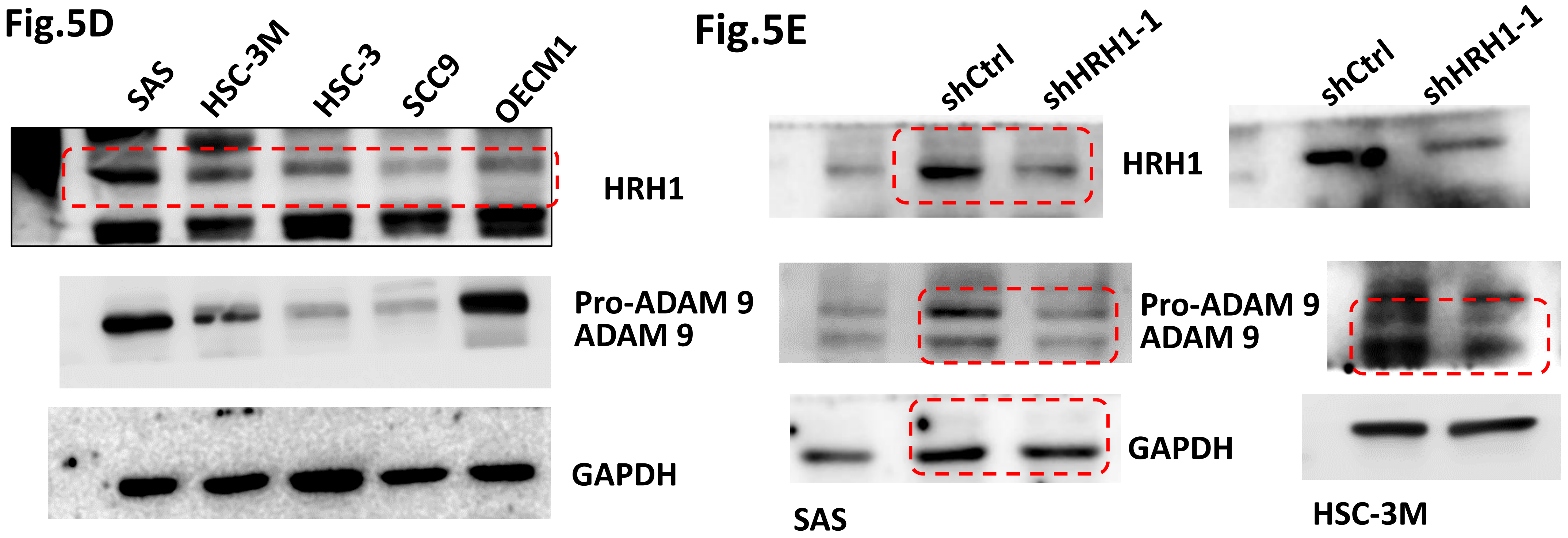
Fig.5D
SAS
HSC-3M
HSC-3
SCC9
OECM1
HRH1
Pro-ADAM 9
ADAM 9
GAPDH
Fig.5E
shCtrl
shHRH1-1
shCtrl
shHRH1-1
HRH1
Pro-ADAM 9
ADAM 9
GAPDH
SAS
HSC-3M

Fig.5H

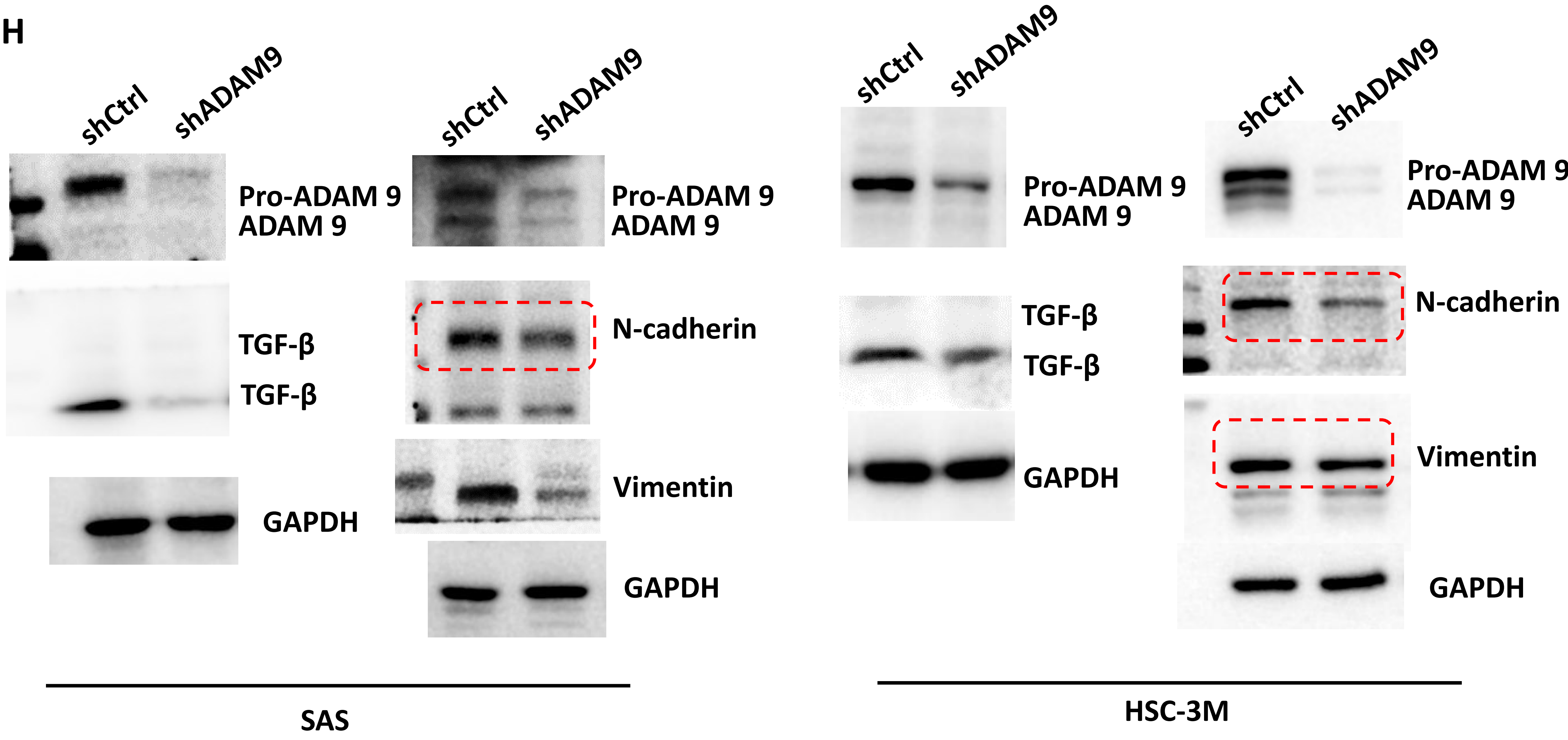

shCtrl
shADAM9
Pro-ADAM 9
ADAM 9
TGF-β
TGF-β
GAPDH
shCtrl
shADAM9
Pro-ADAM 9
ADAM 9
N-cadherin
Vimentin
GAPDH
SAS
shCtrl
shADAM9
Pro-ADAM 9
ADAM 9
TGF-β
TGF-β
GAPDH
shCtrl
shADAM9
Pro-ADAM 9
ADAM 9
N-cadherin
Vimentin
GAPDH
HSC-3M

Fig.5I

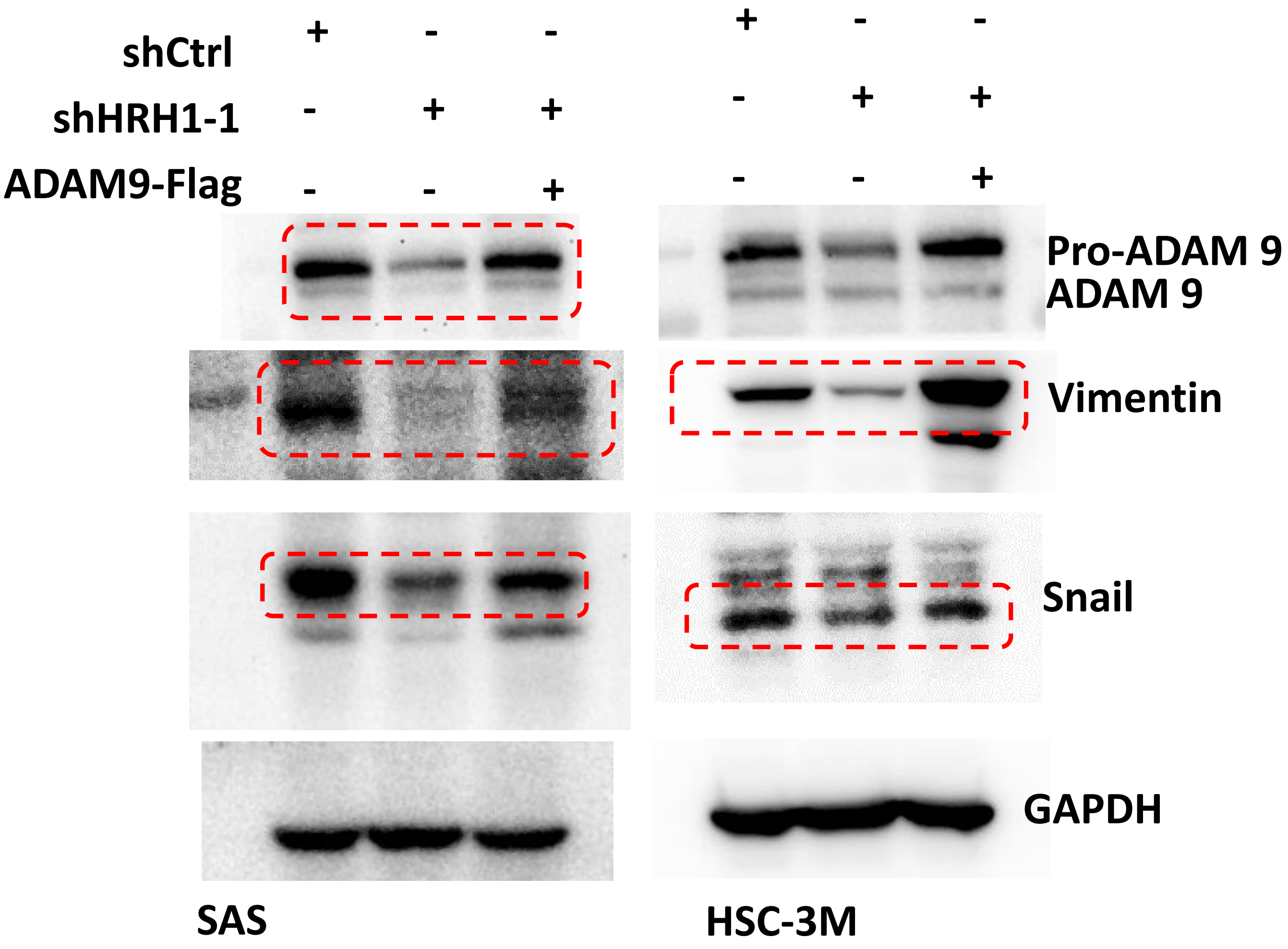
shCtrl + - - + - -
shHRH1-1 - + + - + +
ADAM9-Flag - - + - - +
Pro-ADAM 9
ADAM 9
Vimentin
Snail
GAPDH
SAS
HSC-3M

Fig.5K

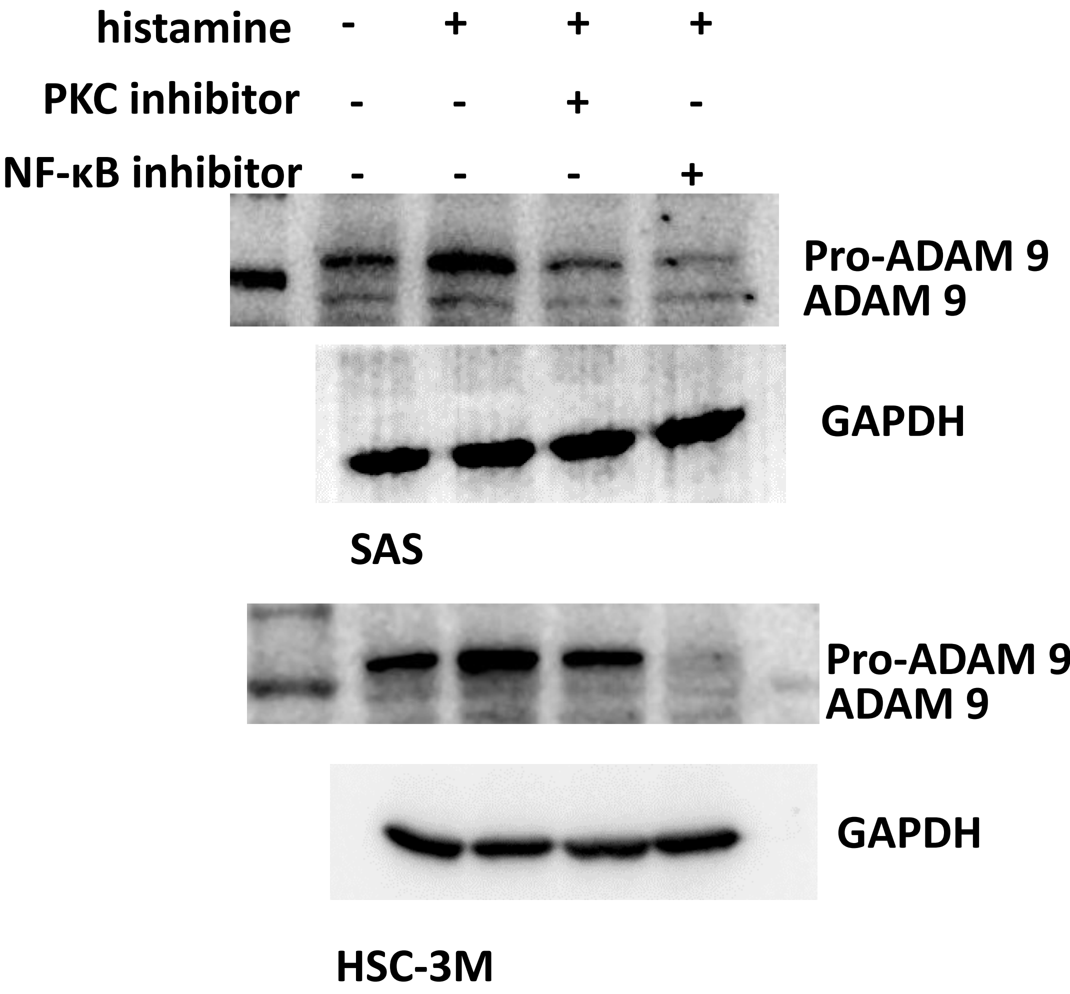
histamine - + + +
PKC inhibitor - - + -
NF-κB inhibitor - - - +
Pro-ADAM 9
ADAM 9
GAPDH
SAS
Pro-ADAM 9
ADAM 9
GAPDH
HSC-3M

Fig.7E

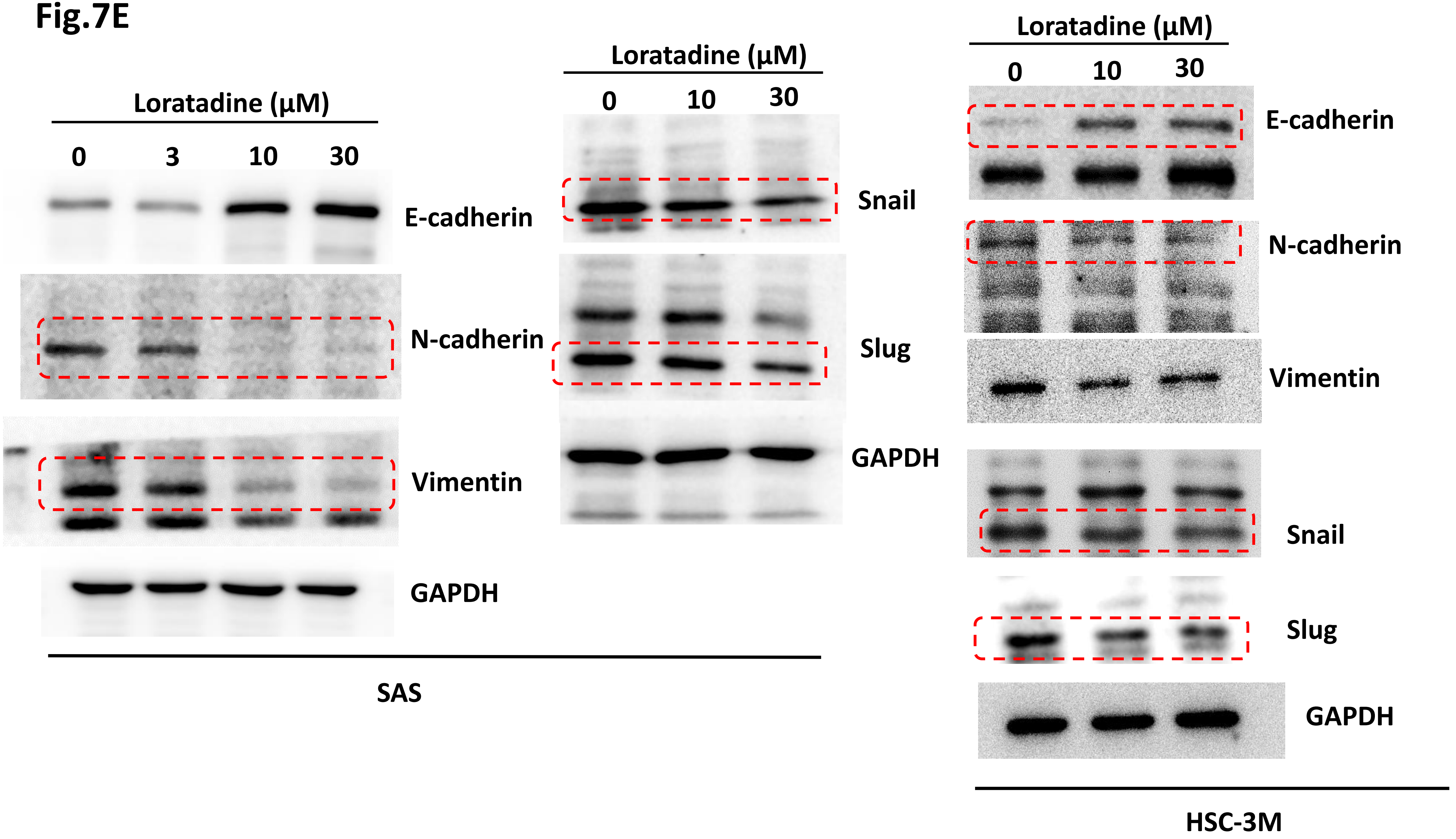
Loratadine (μM)
0
3
10
30
E-cadherin
N-cadherin
Vimentin
GAPDH
Loratadine (μM)
0
10
30
Snail
Slug
GAPDH
SAS
Loratadine (μM)
0
10
30
E-cadherin
N-cadherin
Vimentin
Snail
Slug
GAPDH
HSC-3M

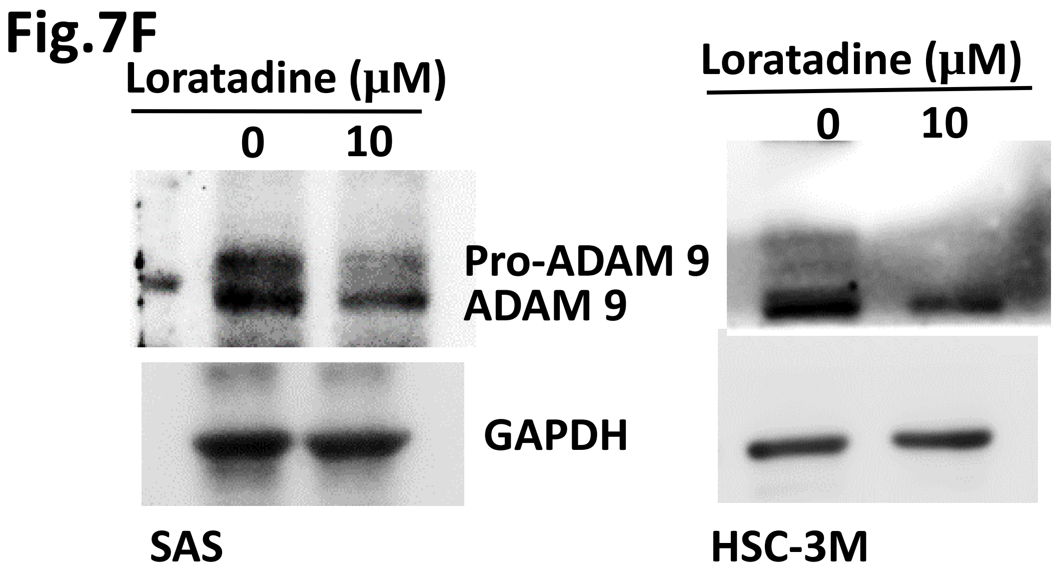
Fig.7F
Loratadine (μM)
0
10
Pro-ADAM 9
ADAM 9
GAPDH
SAS
Loratadine (μM)
0
10
HSC-3M

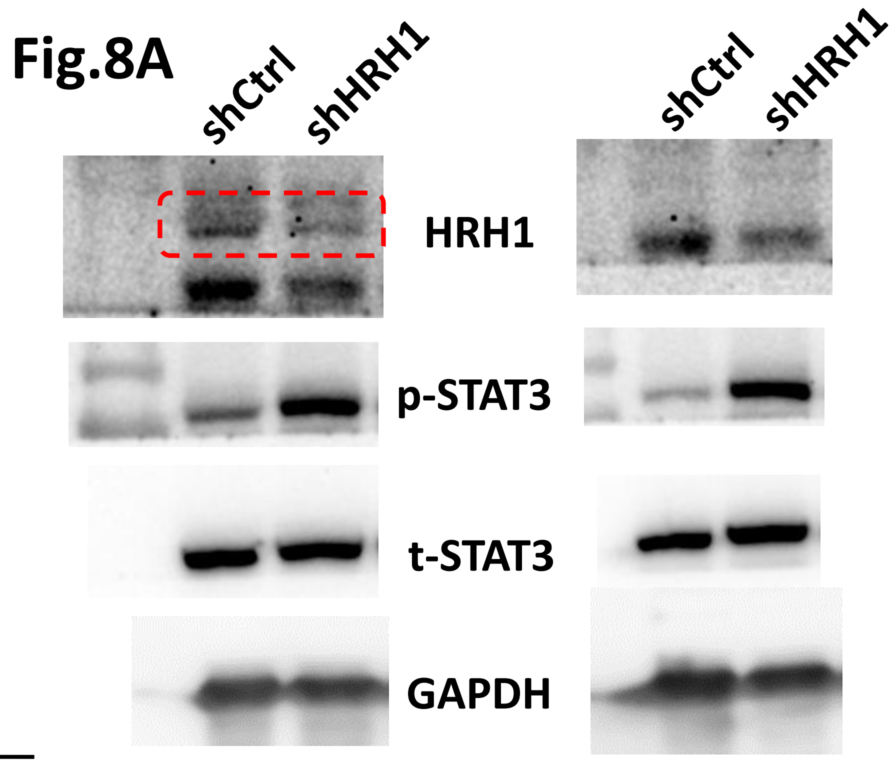
Fig.8A
shCtrl
shHRH1
HRH1
p-STAT3
t-STAT3
GAPDH
shCtrl
shHRH1

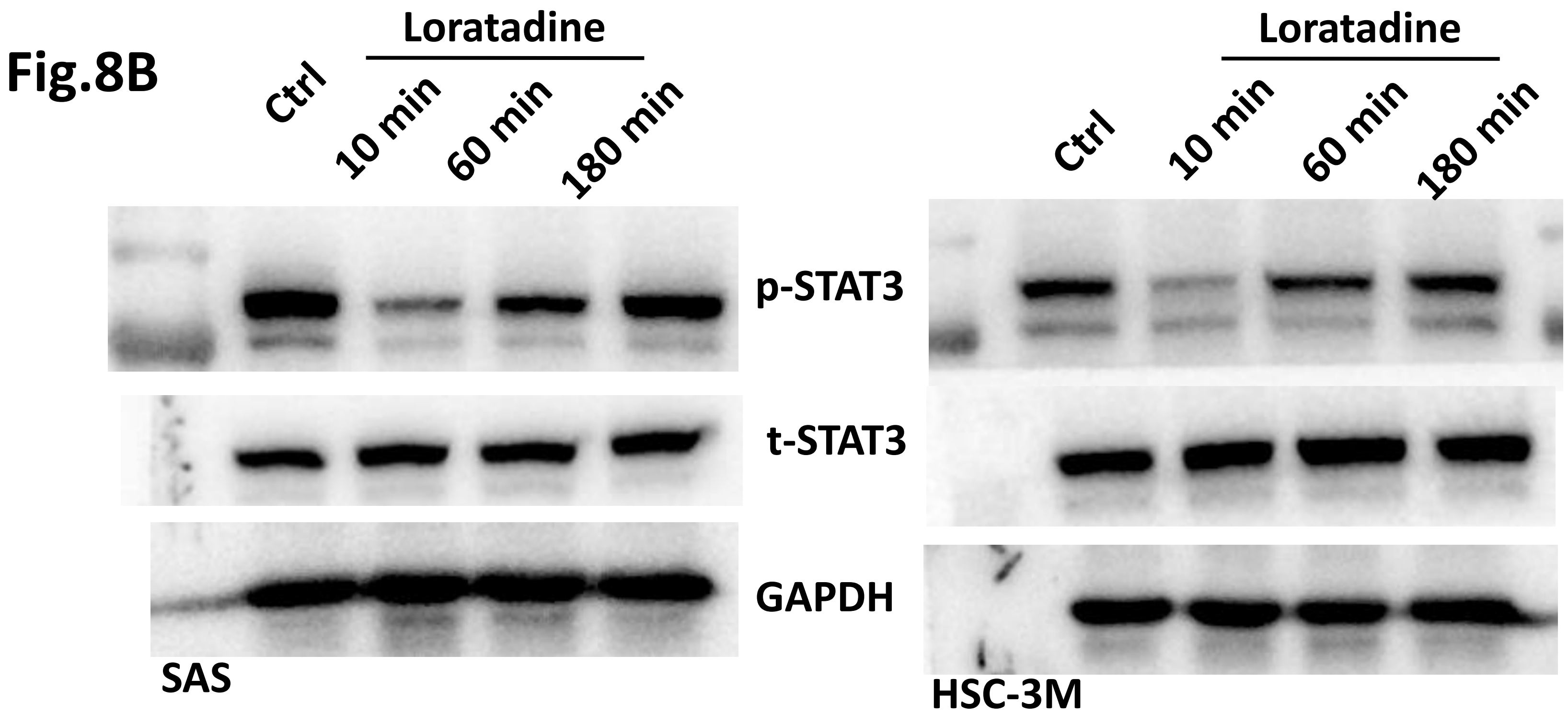
Fig.8B
Loratadine
Ctrl
10 min
60 min
180 min
p-STAT3
t-STAT3
GAPDH
SAS
Loratadine
Ctrl
10 min
60 min
180 min
HSC-3M

Fig.8F

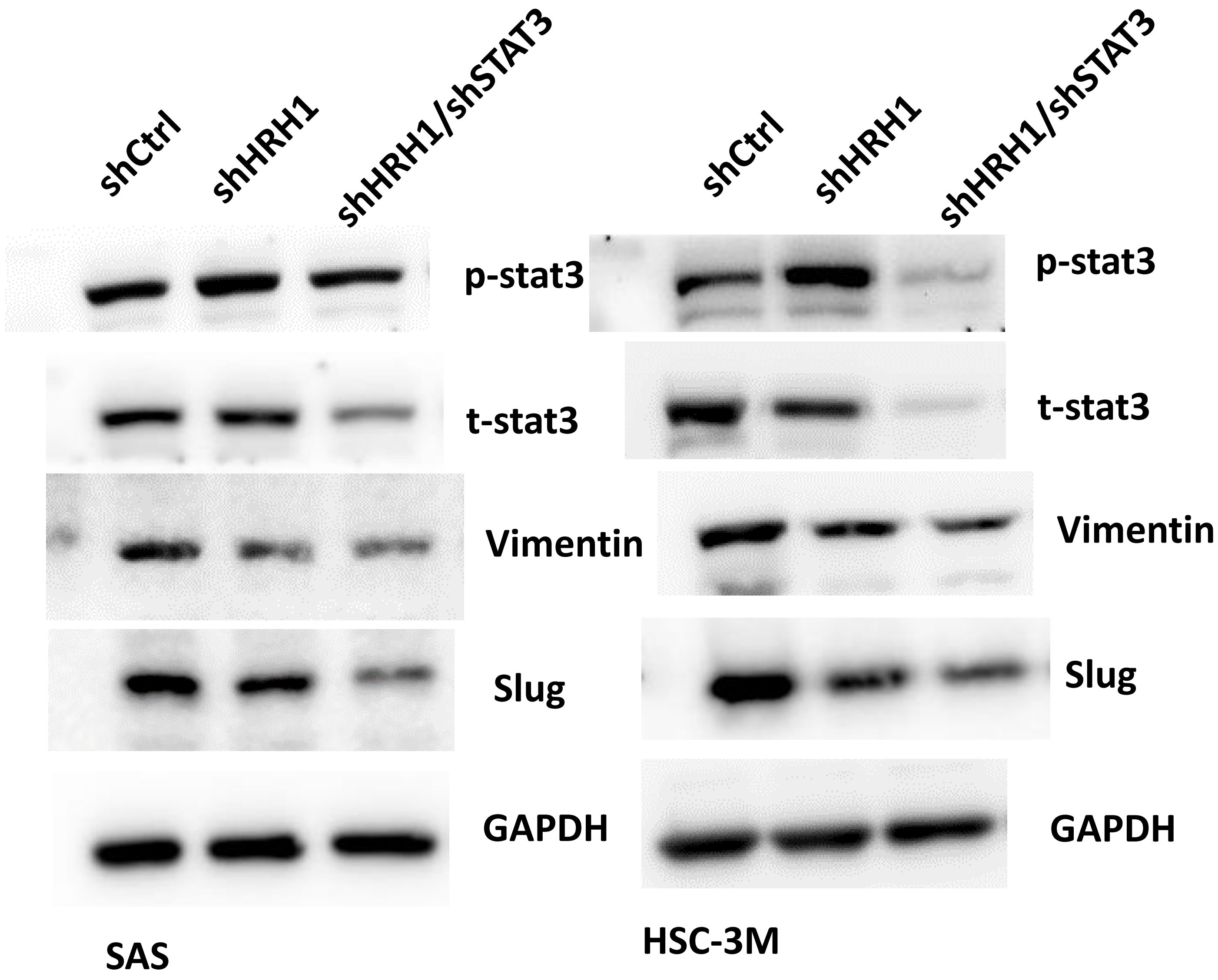
shCtrl
shHRH1
shHRH1/shSTAT3
p-stat3
t-stat3
Vimentin
Slug
GAPDH
SAS
shCtrl
shHRH1
shHRH1/shSTAT3
p-stat3
t-stat3
Vimentin
Slug
GAPDH
HSC-3M

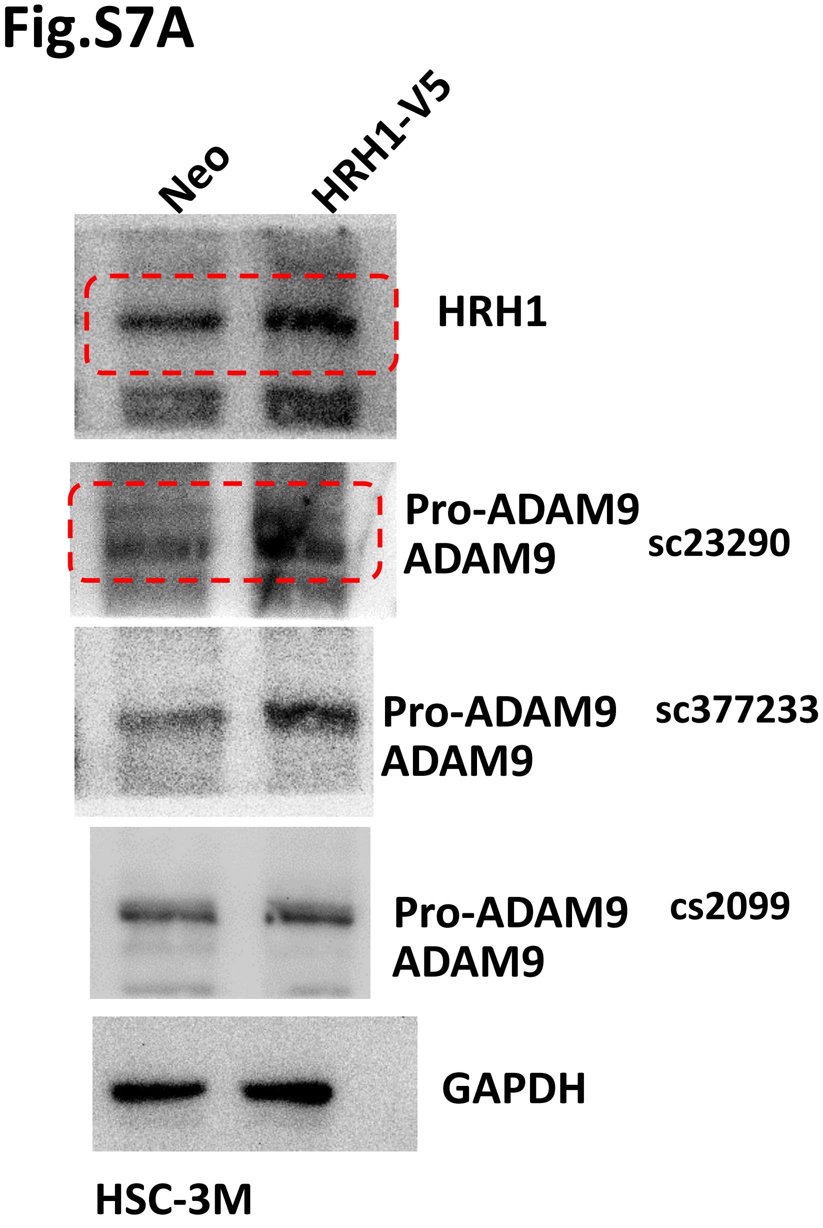
Fig.S7A
Neo
HRH1-V5
HRH1
Pro-ADAM9
ADAM9
sc23290
Pro-ADAM9
ADAM9
sc377233
Pro-ADAM9
ADAM9
cs2099
GAPDH
HSC-3M

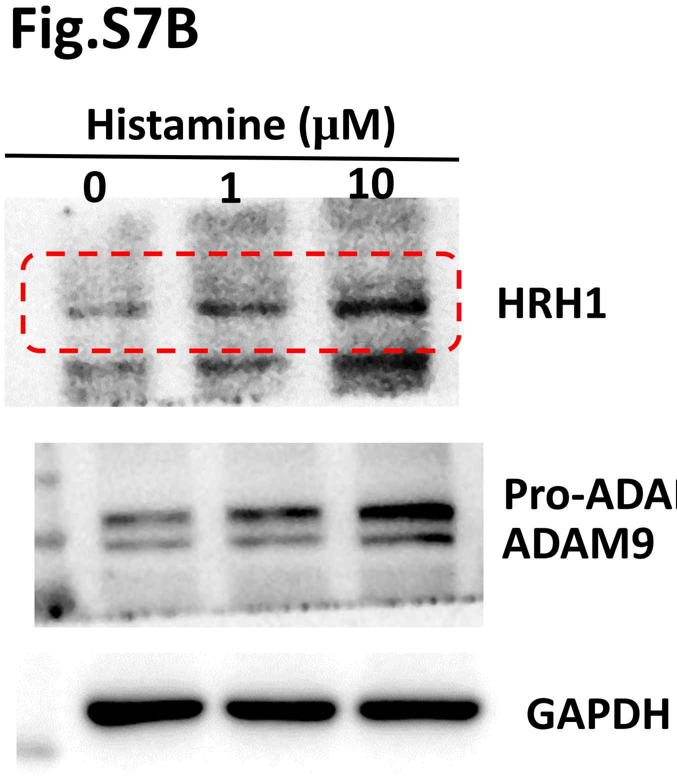
Fig.S7B
Histamine (μM)
0
1
10
HRH1
Pro-ADAM9
ADAM9
GAPDH

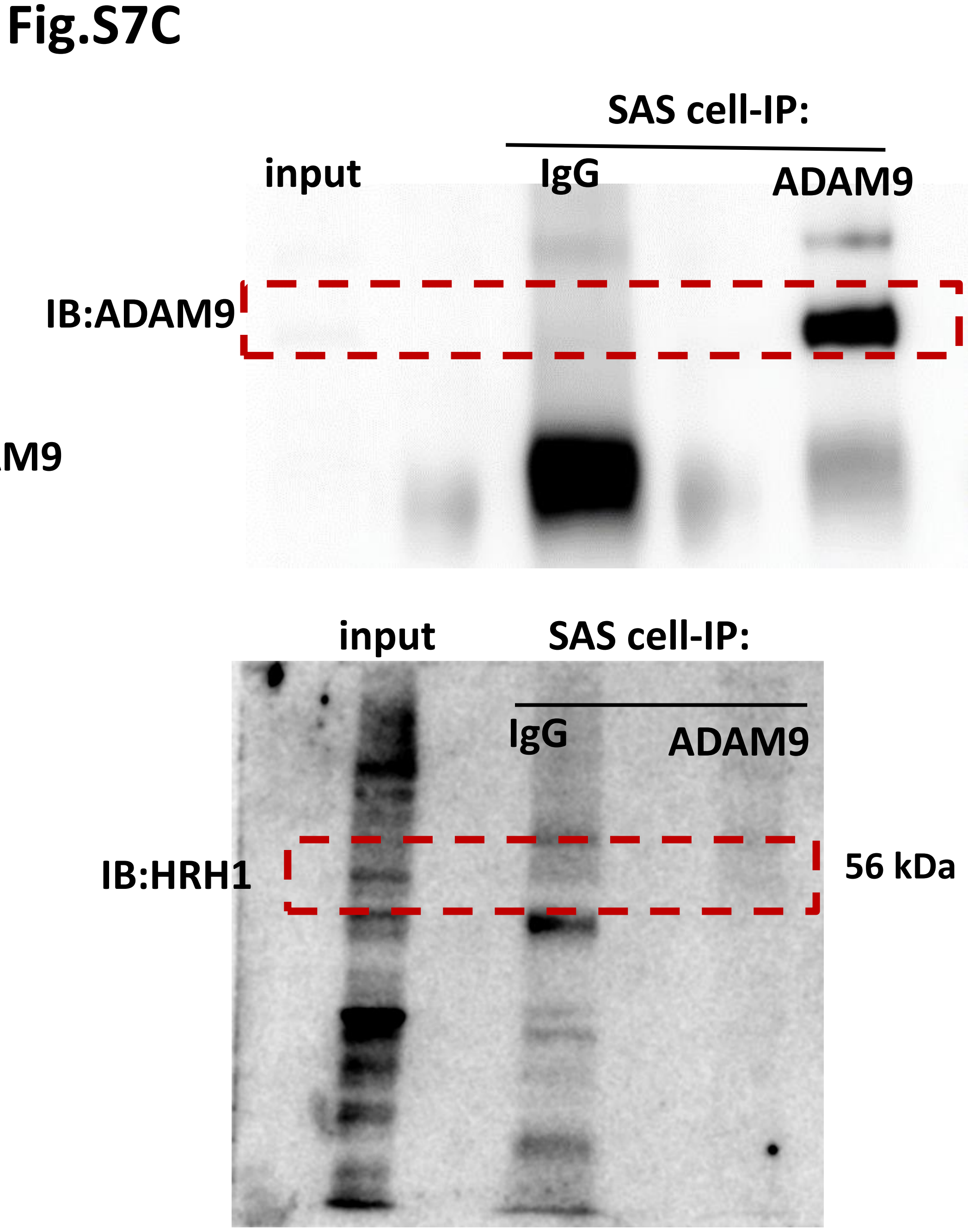
Fig.S7C
SAS cell-IP:
input
IgG
ADAM9
IB:ADAM9
input
SAS cell-IP:
IgG
ADAM9
IB:HRH1
56 kDa

## Fig.S10

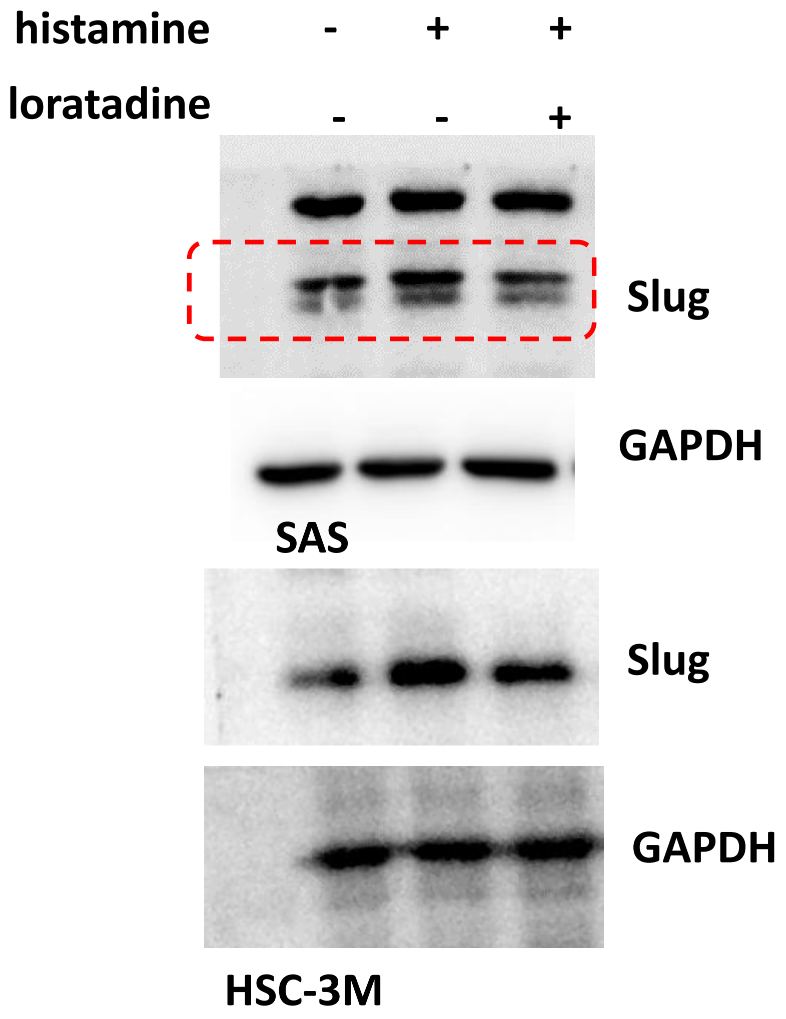

histamine - + +
loratadine - - +
Slug
GAPDH
SAS
Slug
GAPDH
HSC-3M


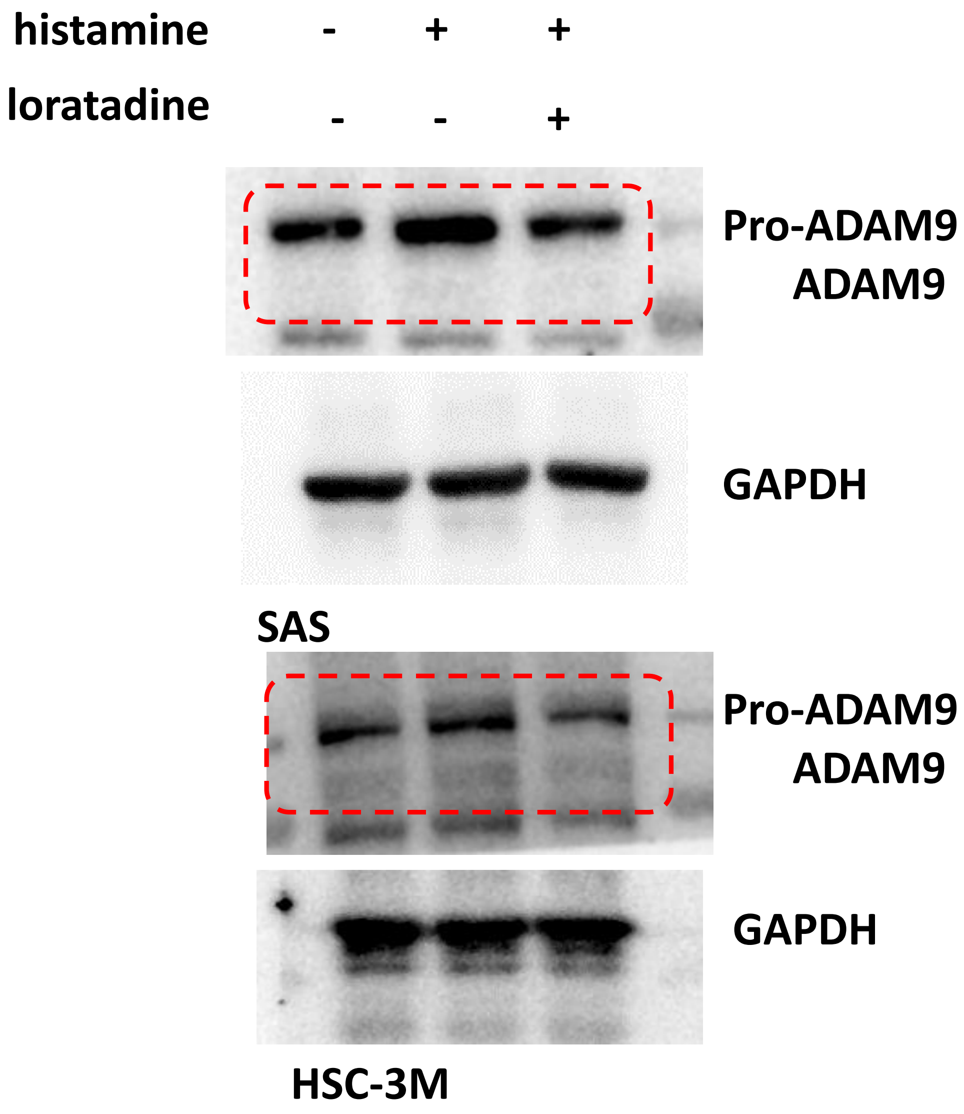

histamine - + +
loratadine - - +
Pro-ADAM9
ADAM9
GAPDH
SAS
Pro-ADAM9
ADAM9
GAPDH
HSC-3M

Fig.S11

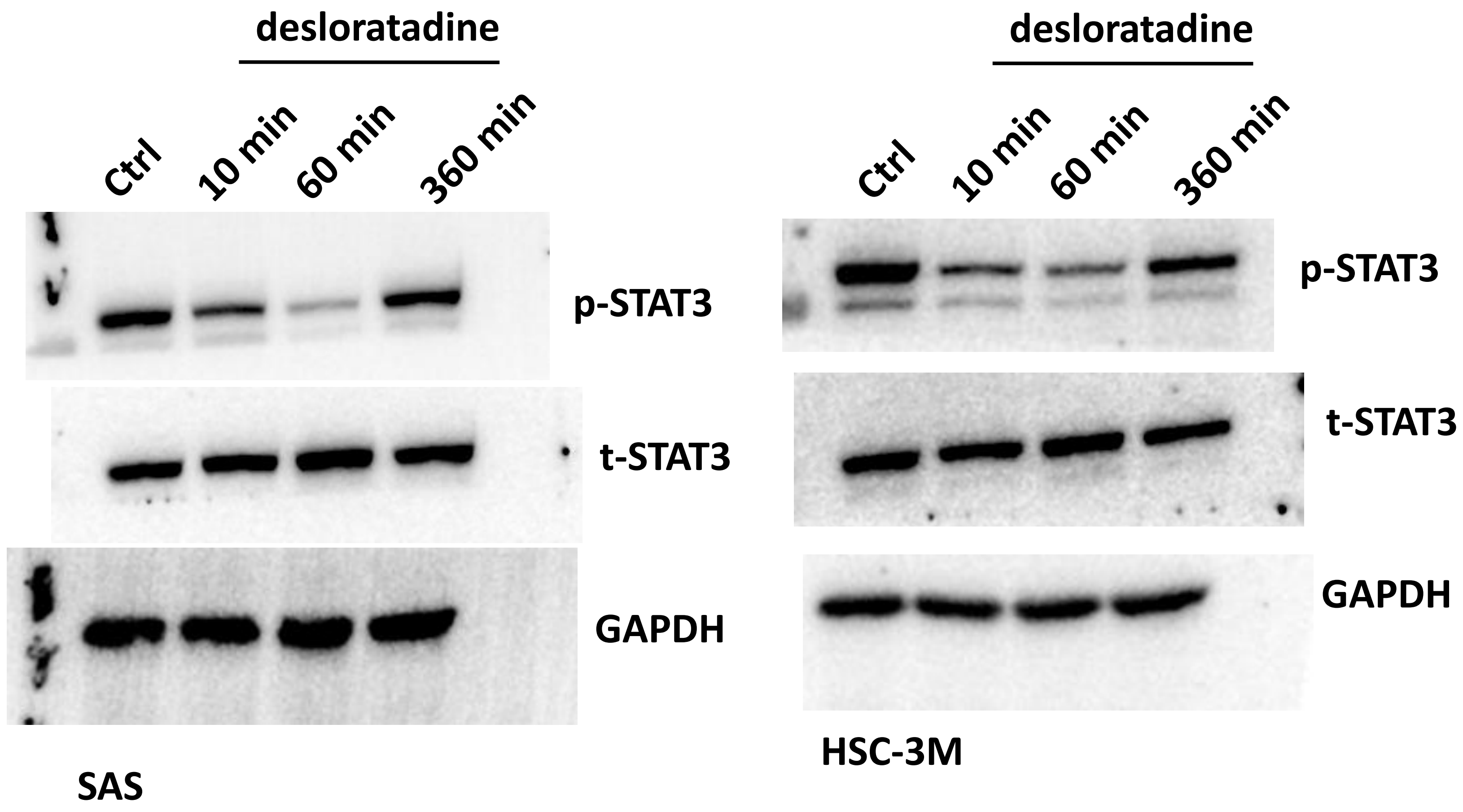
desloratadine
Ctrl
10 min
60 min
360 min
p-STAT3
t-STAT3
GAPDH
SAS
desloratadine
Ctrl
10 min
60 min
360 min
p-STAT3
t-STAT3
GAPDH
HSC-3M

Fig.S15

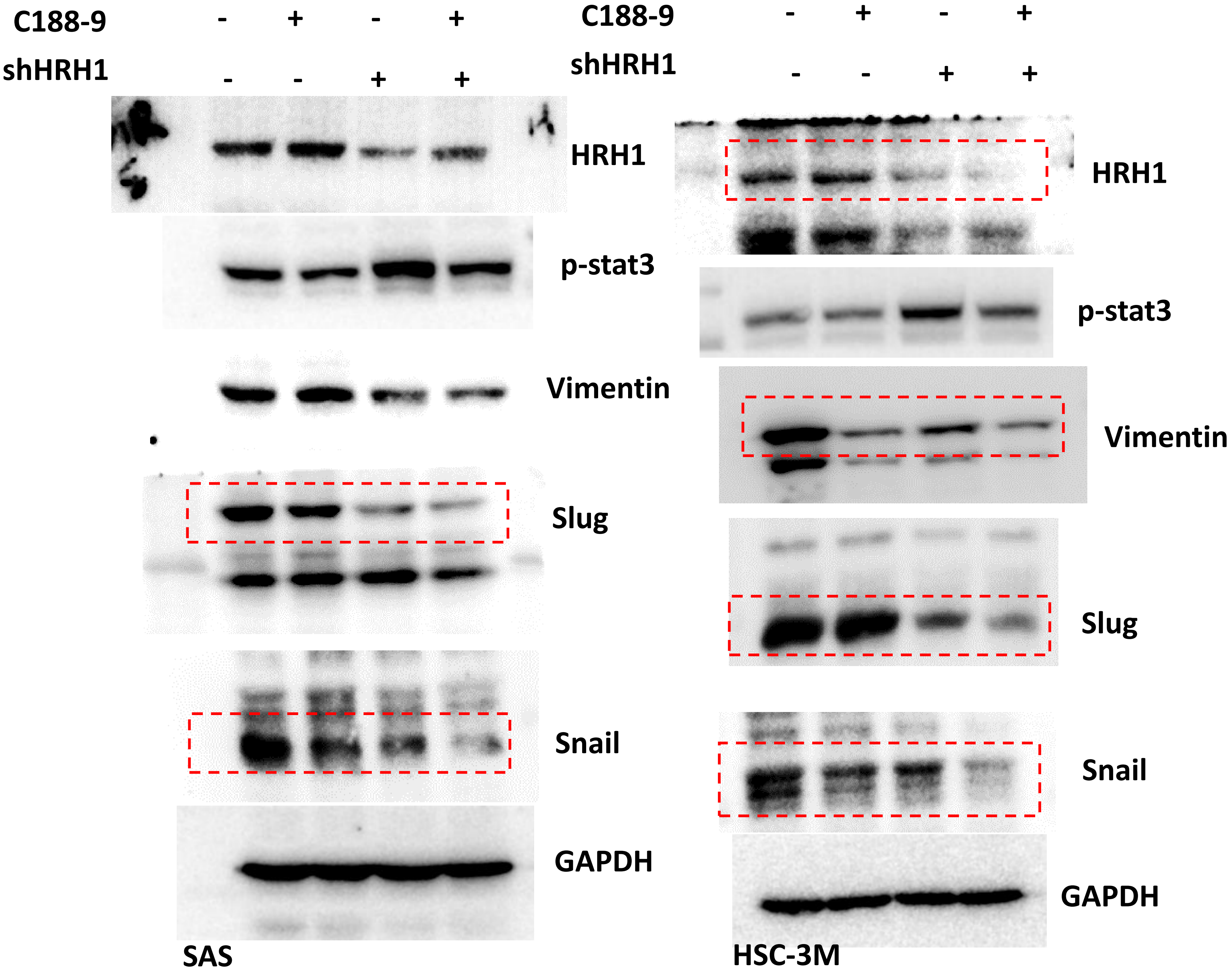
C188-9 - + - +
shHRH1 - - + +
HRH1
p-stat3
Vimentin
Slug
Snail
GAPDH
SAS
C188-9 - + - +
shHRH1 - - + +
HRH1
p-stat3
Vimentin
Slug
Snail
GAPDH
HSC-3M
